# Multivariate analysis applied in dataset of Poison Control Center of São Paulo, Brazil

**DOI:** 10.1038/s41598-020-66485-w

**Published:** 2020-06-11

**Authors:** Sarah Eller, Alexandre Dias Zucoloto, Carolina Dizioli Rodrigues de Oliveira, Edna Maria Miello Hernandez, Ligia Veras Gimenez Fruchtengarten, Flávia Neri Meira de Oliveira, Tiago Franco de Oliveira, Mauricio Yonamine

**Affiliations:** 1 School of Pharmaceutical Sciences, University of São Paulo, São Paulo, SP Brazil; 2 Federal University of Health Sciences of Porto Alegre, Porto Alegre, RS Brazil; 3 Poison Control Center of São Paulo, São Paulo, SP Brazil; 4 Pharmacy College, Federal University of Goiás, Goiânia, GO Brazil

**Keywords:** Liquid chromatography, Epidemiology

## Abstract

Multivariate analysis techniques could be used to identify possible intercorrelations in intoxications cases. The statistical analyses used were a multiple logistic regression, multiple correspondence analysis, principal component and hierarchical cluster analysis. Of the 320 samples analysed, 192 samples were positive for some of the investigated toxic agents, of which 100 were positive for ethanol and 131 were positive for other substances. It was possible to group the patients into 3 clusters, which appears 66.5% of this information in the three first factorial axes. On the first axis, the male patients were separated from the female patients. Patients exposed to drugs, between 30 and 39 years old were grouped in the same cluster. On the second factorial axis, patients who were intoxicated with ethanol and who became intoxicated with diazepam were grouped. This work contributed to the mapping of intoxication cases at the Poison Control Centre of the São Paulo city, Brazil (CCI-SP) and serves as an initial study for the creation of a database that could be updated constantly and thus could provide a toxicovigilance system for educational policies.

## Introduction

Developing intoxication databases is an essential tool in toxicology surveillance programmes. In addition, this type of database is an important tool for the planning, organization, development and evaluation of actions to improve and assist health professionals in emergency toxicological care, as well as for the standardization of related technical activities. The database must be sound and extended to all levels of an organization, including citizens, to trigger actions to reduce and control these risks and effectively prevent them^1,2^.

According to Hair Jr *et al*.^3^, the use of multivariate analysis potentially provides useful information that would not be obtained with only an evaluation between two variables. Hardyck and Petrinovich^4^ published a book stating that multivariate statistical methods would be increasingly explored in the future and that the use of such tools would help researchers in many areas to address their problems and plan research. In recent years, multivariate analysis techniques have been applied in several sectors and adopted in scientific research to verify whether studied variables are correlated. Examples of variables for which these techniques have been used include the classification of leukaemias^5^, the prediction of the outcome after treatment for smokers^6^, statistical probability simulations^7^, the evaluation of chemical variability in plants^8–10^, the classification of adulterants and food composition^11–13^ and the evaluation of organ weights in toxicity studies^14^. However, the use of these analysis techniques is still relatively recent in toxicology. With the application of multivariate analysis in cases of intoxication, it is possible to characterize the events and provide sufficient information to support educational actions related to the prevention of intoxication, as well as to provide advice on the treatments of intoxication and to increase capacity for diagnosing suicidal behaviour in emergency services. For this, one prediction test and three variable correlation tests were selected. The multiple regression analysis is justified by its ability to identify the variables responsible for the severity of poisoning. The analysis of multiple correspondence analysis (MCA) and hierarchical cluster analysis (HCA), are justified by their ability to group patients profiles by the proximity that individuals are in the mathematical space in the graph generated by the MCA and consequently to be grouped due to the similarity of the data of the variables of the individuals^15^.

Therefore, the purpose of this work was to identify and quantify exogenous agents in biological samples from the Poison Control Centre of the São Paulo city (CCI-SP), correlating the data obtained from these analyses with the clinical outcome of the patients, through multivariate analysis and aiming to contribute to the improvement of Brazilian health care services.

## Material and methods

### Sample collection and analysis

The developed method was applied to 320 blood samples collected from patients with suspected poisoning, evaluated at the CCI-SP located in the Hospital Doctor Arthur Ribeiro de Saboya (HMARS), São Paulo, Brazil. Samples were collected between November 2014 and December 2017 from patients with suspected poisoning, subject to toxicological analysis. Generally, blood represents the matrix of choice in clinical toxicological analyses, since pharmacological effects may be correlated to its concentration. During their permanence at the Hospital, if the patient wishes to participate, then a Consent and Informed (IC) term is given out and they are only included in the study upon their permission and respective signature. Patients under the age of 18 and/or at an unconscious state can only be included in the study with the permission of their legal guardian. All samples were analysed after collection and then stored at −20 °C. The study protocol was previously approved by the Bioethics Committee in Medicine of the HMARS (Ethics Protocol Approval No.018/CEM/HMARS - 2014) and by the Research Ethics Committee of the School of Pharmaceutical Sciences of the University of São Paulo (Ethics Protocol Approval No. 902 088). The study procedures were performed in accordance with the ethical standards and all subjects or their parents provided written informed consent prior to study participation.

Blood samples were analysed using liquid-liquid extraction and high‐performance liquid chromatography coupled with a diode‐array detector (Shimadzu® 2030LC 3D; Kyoto, Japan). Drug-free (blank) blood samples used for the development and validation of the method were obtained from volunteers who declared not to have used any substance listed in the study. To verify its negativity, an aliquot of each sample was extracted and analyzed according to the method published previously^16^.

### Statistical analysis

Statistical descriptive frequency analyses and multivariate analyses were performed using the results of quantification of the chemical substances (continuous variable) and the information contained in the charts of hospital care (nominal or categorical variable). The statistical analyses used in this work were the multiple logistic regression analysis (MLRA), MCA, PCA and HCA, also called cluster analysis^15^.

The matrix was formed by variables in the columns and individuals in the rows. The variables studied were age, sex, chemical substance, type of event (intoxication, exposure, adverse reaction, or differential diagnosis), route of exposure (oral, cutaneous, respiratory, parenteral, ocular, rectal, or vaginal), type of use (occupational, accidental, environmental, therapeutic use, inadequate prescription, misuse, or miscarriage induction), type of exposure (acute single, acute repeated, chronic, acute over chronic, or ignored), type of effect (general, neurological/psychic, gastrointestinal, respiratory, cardiovascular, renal, or cutaneous), case development (cure without sequelae, cure with sequelae, death due to intoxication, death from another cause, or ignored) and xenobiotics concentration.

To perform the statistical treatment of the data, the variables were self-staggered, thus providing different weights and allowing a comparison among the variables. After the data treatment, the results were expressed through a dendrogram. In the vertical dendrogram, the interpretation is performed from right to left, where the *y*-axis indicates the distances between the formed groups; the *x*-axis represents the groups joined in descending order of similarity. For the analysis, values of *p* < 0.05 were accepted. The statistical software used was the *Système Portable d’Analyse (SPAD) Coheris* (2010), professional version, and SAS, Cary, NC (1996).

### Multiple logistic regression analysis

MLRA was used to evaluate the dependent variable and predict changes as responses in the independent variables. The dependent variable was “evaluation of cases”, which was recoded into two categories, more severe and less severe. The independent variables were the continuous or categorical. The MLRA was qualified by a linear model that establishes a method based on Likelihood research. It was estimated by the “odds ratio” with a 95% confidence interval^3^, which indirectly calculates the relative risk. The calculated model established a predictive situation measured by the value of *r*^2^.

### Hierarchical cluster analysis

The similarities among the samples were evaluated by HCA, based on the distribution of the variables within the graphic of MCA. The nearest-neighbour technique was applied by the Benzécri algorithm^17^ to verify this similarity, and the hierarchical groupings were formed according to Ward’s minimum variance method^18^.

### Multiple correspondence analysis and principal component analysis

With MCA, it was possible to stipulate correspondence between variables and individuals. From the results of these analyses, it was possible to graphically visualize the relations among a large data set, that can reveal correlation tendency. The PCA was also performed as a complementary analysis. PCA is widely used to reduce the dimensionality of a large number of interrelated variables while retaining as much of the information as possible without a significant loss of information. Therefore, PCA was used to establish the interrelationships between the variables, by multiplying the original matrix with the transposed matrix. The MCA was performed with all the categorical and continuous variables, but for the categorical variables, a value of *p* < 0.05 was considered.

### Canonical correlation analysis

Canonical correlation analysis (CCA) correlated several independent and dependent variables by the SAS CANCOR. The predictive ability was evaluated by a canonical redundancy analysis (SAS, 1996). The Tukey test was used to compare the concentrations of the substances. A value of *p* < 0.05 was considered^3^.

## Results

The analytical methodology was validated according to international guidelines and recommendations for validation of analytical methods, by establishing parameters such as the lower limit of quantification (LLOQ), linearity, accuracy, precision, selectivity and recovery rate. The developed and validated analytical method were previously described and published by our research group^16^.

Considering the toxic agent and the evaluation of intoxication, the most observed cases were mild intoxication (189 cases) correlated with drugs of abuse (71%; 135 cases). Considering the moderate intoxication (55 cases), drugs of abuse (55%; 30 cases) and medicines (25%; 14 cases) were main toxic agents responsible for this type of intoxication. Severe intoxication (11 cases) was caused by medicines and drugs of abuse, both of which accounted for 36% of the cases. Suicide attempts (75 cases) occur mainly with the use of medicines (64%; 48 cases), as previous reported^15,17,18^. In addition, a greater number of suicide attempt involved mild intoxication (49.3%; 37 cases), with only 8% of the cases (6 cases) considered severe. Approximately 68% of the cases involved substance abuse (136 cases of the total 200 cases) and were considered mild intoxication, and 15.5% (31 cases) were considered moderate. Only 2% (4 cases) of these cases were considered severe intoxication.

The MLRA, with the dichotomous variable, obtained a *p* value <0.0001 by the Likelihood ratio test. The R^2^ values of the obtained model were 0.324 for Cox and Snell and 0.387 for MacFadden’s pseudo, indicating a predictive power higher than 30%. The data were also evaluated through an MLRA, and the dependent variable was the evaluation of the cases. According to patient medical records, the “evaluation” can be classified into six types of intoxication: non-toxic, probably non-toxic, intoxication not excluded, mild, moderate and severe. Logistic regression (*y*) is dichotomous; therefore, this classification was reduced to only two categories of intoxication, with the first four types considered “less severe” and the last two types considered “more severe”. It is observed that the variable “evaluation” of the most severe cases is explained by the variables occurrence (differential diagnosis and exposure), circumstance (ignored), exposure (nasal and oral), type (repeated acute, single acute and chronic), toxic agent (drugs, rodenticides) and the substance cocaine, confirmed by the “Odds ratio”, as shown in Table 1. In bold the variables and categories that explain the variable evaluation of the most severe cases.

**Table 1:** Odds Ratio of the Multiple Logistic Regression (95% confidence interval). In bold the variables and categories that explain the variable evaluation of the most severe cases.

Variables	*Category*	*Odds ratio*
Age		1.021
Gender	Female	0.564
Occurrence	Differential diagnosis	0.055
Occurrence	Exposure	0.041
Circunstance	Abuse	1.204
**Circunstance**	**Abuse_ Suicide attempt**	**15.495**
**Circunstance**	**Ignored**	**17.670**
Circunstance	Other	7.306
Exposure	Ignored	1.046
Exposure	Nasal	0.096
Exposure	Oral	2.150
Exposure	Oral_Nasal	0.195
Exposure	Oral_Respiratory	1.561
Exposure	Oral_Respiratory_Nasal	0.125
Type	Pepeated acute	0.214
Type	Acute on chronic	2.094
Type	Single acute	0.119
Type	Single acute _ Acute on chronic	0.017
Type	Chronic	0.058
Toxic agent	Pesticide	5.965
Toxic agent	Ignored	0.832
Toxic agent	Medicines_Drugs of abuse	0.785
**Toxic agent**	**Medicines_Pesticides**	**28.813**
Toxic agent	Pesticides	9.981
Evolution	Cure	3.142
Evolution	Unconfirmed cure	5.322
Evolution	Ignored	1.599
**Evolution**	**Death**	**39.468**
Sertraline		8.369
Phenobarbital		1.077
Diazepam		3.677
Carbamazepine		0.824
Phenytoin		0.978
**Cocaine**		**23.862**
Acetaminophen		0.857

**Figure 1:**
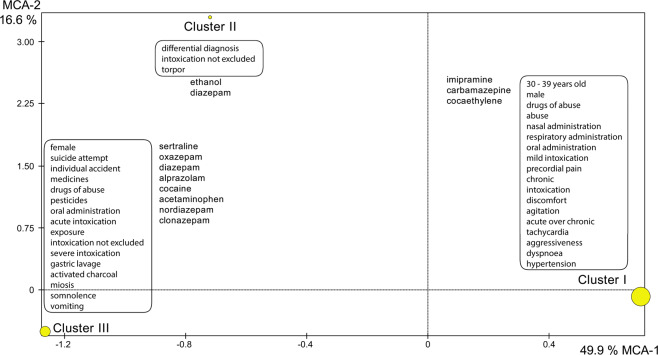
Graphical representation of multiple correspondence analysis of all variables with 3 clusters.

**Figure 2:**
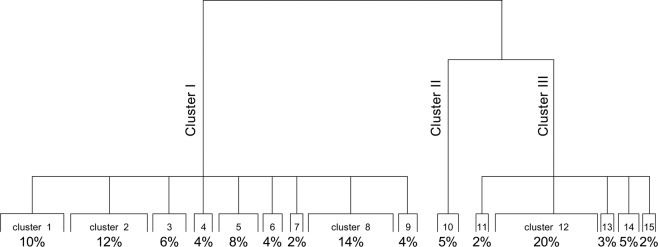
Dendrogram of Hierarchical Cluster Analysis (HCA), grouping individuals into 3 and 15 clusters.

The correlation between the variables was verified through the MCA. The statistical software SPAD, version 7.4 (Coheris, France), was used to evaluate the data. The similar characteristics of the patients were grouped in three and fifteen clusters. The division into three clusters is more important than the division into fifteen because the former has lower *p* values; however, both divisions were grouped within the stipulated level of significance^3^. In the Table 2 it is showed the important variables used by the HCA, considering a division into three clusters. The formation of each cluster occurred due to the similarity obtained from the variables described in the table. In Fig. 1, the MCA explains 66.5% of the total variation, with principal component (PC) 1 accounting for 49.9% and PC 2 accounting for 16.6% in the data set. Analysing the dendrogram with 15 clusters, the largest number of individuals is grouped in clusters 12 (20%), 8 (14%), 2 (12%) and 1 (10%) (Fig. 2). In the CCA, no acceptable correlation was observed between the dependent and independent variables evaluated. The R values found were lower than 0.4, indicating low predictive power. The PCA was also performed as a complementary analysis, and it obtained 26.35% of the information in the first two axes.

**Table 2:** Description of the important variables used by the HCA, considering a division into 3 clusters. The formation of each cluster occurred due to the similarity obtained from the variables described in the table.

*Cluster*	Variables	Data	*p* value
I	Substances	Imipramine	0.269
		Carbamazepine	0.284
		Cocaethylene	0.495
	Toxic agent	Drugs of abuse	0.0001
	Circunstance	Abuse	0.0001
	Exposure	Oral	0.0001
		Nasal	0.0001
		Respiratory	0.0001
	Gender	Male	0.0001
	Type	Acute repeated	0.0001
		Chronicle	0.0001
	Evaluation	Mild intoxication	0.0001
	Cardiovascular manifestations	Chest pain	0.0001
		Hypertension	0.022
	General manifestation	Unwell	0.002
	Neurological manifestations	Agitation	0.002
		Aggressiveness	0.011
	Respiratory manifestation	Dyspnea	0.012
	Age	30 to 39 years old	0.007
	Occurrence	Intoxication	0.0001
II	Substance	Ethanol	0.89
		Diazepam	0.320
	Occurrence	Differential diagnosis	0.0001
	Evaluation	Poisoning not excluded	0.0001
	Neurological manifestations	Torpor	0.0001
	Exposure	Ignored	0.0001
II	Toxic agent	Ignored	0.0001
	Type	Ignored	0.0001
	Circumstance	Ignored	0.0001
III	Substance	Sertraline	0.014
		Oxazepam	0.046
		Diazepam	0.060
		Alprazolam	0.116
		Cocaine	0.175
		Acetaminophen	0.260
		Nordiazepam	0.304
		Clonazepam	0.374
	Circumstance	Suicide attempt	0.0001
		Individual accident	0.001
	Exposure	Oral	0.0001
	Toxic agent	Medication	0.0001
		Drugs of abuse	0.002
	Type	Single acute	0.0001
	Genre	Feminine	0.0001
	Occurrence	Exposure	0.0001
	Evaluation	Poisoning not excluded	0.001
		Severe intoxication	0.025
	Neurological manifestations	Somnolence	0.005
		Miosis	0.005
		Agitation	0.0001
	Gastrointestinal manifestation	Vomiting	0.007
	Treatment	Gastric lavage	0.0001
		Single dose activated carbon	0.0001

According to Fig. 1, the first axis shows the separation of the female patients who were intoxicated or exposed to medicines, drugs of abuse and pesticides, including suicide attempts, with signs and symptoms of drowsiness, miosis and vomiting and with non-excluded and severe intoxication and the separation of male patients aged 30 to 39 years who were intoxicated with drugs of abuse, with symptoms of aggressiveness, tachycardia, agitation, dyspnoea, hypertension and precordial pain, with mild intoxication. The relationship between being female and drug intoxication is already described in the literature^19^. On the second factorial axis (Fig. 1), we can observe the patients intoxicated with ethanol only, along with patients who became intoxicated with diazepam. It is also possible to observe in the same factorial axis, the separation of the patients with positive results for cocaine (cluster III) and cocaethylene (cluster I) in the toxicological analyses of the other patients (cluster II) where the barycentre is located in the bottom of the MCA chart.

**Figure 3:**
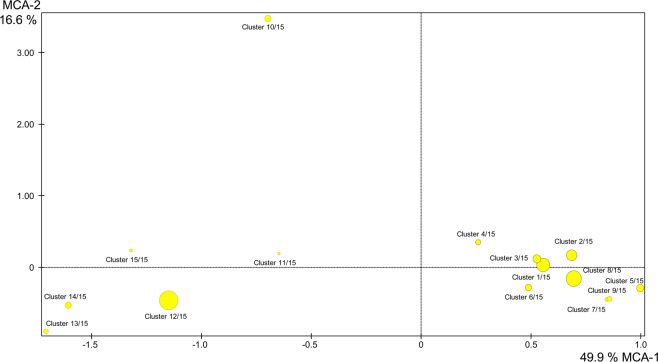
Graphical representation of multiple correspondence analysis of all variables with 15 clusters.

In Fig. 3, all the information regarding the 15 clusters is detailed. The data are the same as described in Fig. 1. However, patients and variables are detailed with 15 clusters.

## Discussion

The objective of this work was to use big data or multivariate analysis methodologies in data obtained from patient’s intoxication suspects. These data are extensive, both in the number of variables collected and in the number of individuals attended at Toxicological Information Centres. Methodologies are often used in large databases in economics, politics, marketing, etc. Its use is not yet observed in the data of intoxicated patients. The analyses aim to evaluate the characteristics of intoxicated patients and which are the factors that potentiate intoxication. The coding of data in continuous or categorical restricts the choice of the statistical method. For correlation analysis by matrix multiplication, we selected PCA to evaluate continuous data and MCA for categorical data. The principle of the two analyses is the same; however, PCA evaluates current data that have innumerable possibilities of answers, whereas in MCA it evaluates fixed data with smaller number of possibilities of answers. Although MCA must transform categorical data into continuous data, subtracting each data with the standard deviation and dividing with the mean of the numerically recoded data variable, generating a fractional data as close as possible to the continuous data. The results of both analyses tend to give the same result. If both tests could use the same matrix, without changing any kind of data coding, the result would be the same. So to try to observe similar trends in both tests, we selected the least demanding *p* value for PCA. The PCA results correspond to a portion of the MCA results. The correlations presented in the PCA also appear in the MCA; however, MCA presented the largest number of possible correlations, even reducing the p value requirement and consequently the significance level.

The descriptive analysis of the frequency considering “evaluation” as a dependent variable showed that the substances detected in blood with the highest frequency in the most severe cases were sertraline, phenobarbital, amitriptyline, diazepam and cocaine. In addition, the severe cases are also explained by the following variables: occurrence (differential diagnosis and exposure), circumstance (ignored), exposure (nasal and oral), type (repeated acute, single acute and chronic), possible toxic agent (rodenticides and cocaine), confirmed by the odds ratio.

Analysing each of the fifteen clusters (Fig. 3), it was verified that in the first group (representing 10% of the patients), for individuals with positive results for chronic cocaine use, through oral and nasal routes, the main symptom observed was tachycardia. The individuals grouped in cluster 2 had similar exposure “abuse”. Cluster 3 separated the male individuals with positive results for cocaine, cocaethylene and ethanol, and the main symptoms presented were aggressiveness and agitation. Patients in cluster 4 were grouped by symptoms of mental confusion, aged between 50 and 59 years old, using sertraline and carbamazepine. Cluster 5 separated the individuals who presented with the highest number of symptoms such as discomfort, dyspnoea, chest pain, dizziness, dehydration, agitation, and tachypnoea. Individuals between 10 and 19 years old who presented abdominal pain, headache and dizziness as symptoms were grouped in cluster 6. Individuals who presented hypertension, tachycardia and palpitations were included in cluster 7, together with the suspect of the use of drugs. In cluster 8, similar individuals in terms of being male, using of drugs of abuse, and reporting precordial pain and palpitations, with a final evaluation of mild intoxication and positives results for ethanol were grouped. Individuals who had acute, chronic abuse of drugs by oral and nasal routes were grouped in cluster 9, and this group presented paraesthesia and anxiety as the main symptoms. Individuals grouped in cluster 10 presented ethanol and diazepam in the toxicological analyses. The assessment was determined as non-excluded intoxication. Additionally, in this cluster, individuals with differential diagnosis and torpor with unknown condition, exposure, type and toxicant were included. In cluster 11, individuals with not-excluded intoxication were included; however, the circumstances around exposure were accidental, and tachypnoea was a symptom. Cluster 12 (20%) included the largest number of positive substances in the toxicological analyses, such as diazepam, oxazepam, and clonazepam; nortriptyline, sertraline and amitriptyline; phenobarbital; cocaine; and ethanol. In this cluster, female patients, with oral intake, acute intoxication and attempted suicide, were also included. These results are in agreement with the literature that reports a higher frequency of intoxication is caused by antidepressants and anticonvulsants^20^. In addition, the use of medicines in suicide attempts is common, especially among women, and antidepressants are often used for this purpose, accounting for approximately 20% of total suicides in the United Kingdom and 20–30% of non-fatal overdoses in the same country^21,22^.

Cluster 13 was characterized by female patients, between 10 and 19 years old, that used medicines including acetaminophen, in attempted suicides. The common route of exposure was oral, which is acutely unique. Additionally, in this group, the treatment used was gastric lavage and activated charcoal. Continuing to analyse cluster 13, the data obtained corroborate the scientific literature. Acetaminophen intake is considered one of the causes responsible for episodes of overdose and acute liver failure in children and adolescents^23^. In the United States, acetaminophen paediatric ingestion accounts for approximately 30,000 reports annually from the National Intoxication Data System^24^. According to published studies evaluating the use of N-acetylcysteine (NAC) and the severity of hepatotoxicity, the higher incidence of hepatotoxicity is directly related to the long time that elapsed between intoxication and administration of NAC. According to Cristoforo and Rahman^23^, only 2% to 7% of patients treated within 8 to 10 hours of drug intake developed severe hepatotoxicity, unlike 26% to 53% of those treated 10 to 24 hours after ingestion. Although the data obtained in the present study are not considered new, they support decision making about the treatments used in poisoning.

Cluster 14 grouped individuals who were between 50 and 59 years old for whom pesticide use, suicide attempted, miosis was the main cause of symptoms, and intoxication was considered moderate. In the univariate study reported by Bucaretchi *et al*.^25^, severe intoxication with the use of pesticides, mainly aldicarb, was reported for suicide attempts in Brazil. Our data also were consistent with these findings with the predominance of these cases occurring in the age group of 50 and 59 years. The individuals between 40 and 49 years old were grouped in cluster 15 (2%) and were the only ones who presented symptoms of respiratory failure, tachypnoea and coma, with considered as severe intoxication. Despite the non-identification of the substances used in common in this group, we can consider that the rapid recognition of these symptoms and their treatment may be crucial for a good prognosis for the patients. In relation to individuals with toxic concentrations, we also verified that individuals with substances at toxic concentrations were grouped in clusters 2, 8 and 12, with clusters 2 and 8 being separated from cluster 12 by the type of toxic agent, being drug of abuse (2 and 8) and medicines (12). Considering the ethanol-positive samples, all individuals who presented blood alcohol concentrations higher than 3.0 g/L were present in the upper part of the second factorial axis of the graph and were grouped in clusters 2, 3 and 10.

Although we tried to incorporate the largest number of complete data, some information was missing from the attendance form. But few variables were missing within each case. If these data could be added to the multivariate analysis, better results could be obtained. This fact reinforces the need to complete the Hospital Care Form in full, as the absence of some essential information did not allow the establishment of certain correlations.

The results of this study show that through the applied multivariate analysis, it was possible to evaluate the relationship of all variables studied among themselves and their influence on individuals. We demonstrated that the cases of intoxication presented at the CCI-SP were classified into three clusters. There was also a correlation among the female gender, medicines use and suicide attempt, with moderate and severe intoxication, and a separation of this group from that of male patients aged between 30 and 39 years who became intoxicated with drugs of abuse and mild intoxication. Health information should be based on data and records whose safety and reliability are guaranteed and that serve as a basis for designing educational and social strategies to prevent poisoning.

## Conclusion

This work contributed to the mapping of intoxication cases addressed by the CCI-SP and was an initial study related to the creation of a database that could be updated constantly and thus provide a toxicovigilance system with a foundation for educational policies.

## Supplementary information

Supplementary material S1.
