# Supplementary material for: Multivariate analysis applied in dataset of Poison Control Center of São Paulo, Brazil
Source: Sci Rep. 2020 Jun 11;10:9498. doi: 10.1038/s41598-020-66485-w (PMC7289857; doi:10.1038/s41598-020-66485-w)
Supplement: Supplementary file 1 — Supplementary material S1. [file 41598_2020_66485_MOESM1_ESM.docx]

**Multivariate analysis applied in dataset of Poison Control Center of São Paulo, Brazil**

Sarah Eller^a,b,^*; Alexandre Dias Zucoloto^a,c^; Carolina Dizioli Rodrigues de Oliveira^c^; Edna Maria Miello Hernandez^c^; Ligia Veras Gimenez Fruchtengarten^c^; Flávia Neri Meira de Oliveira^d^; Tiago Franco de Oliveira^b^; and Mauricio Yonamine^a^

^a^School of Pharmaceutical Sciences, University of São Paulo, São Paulo – SP/Brazil

^b^Federal University of Health Sciences of Porto Alegre, Porto Alegre – RS/Brazil

^c^Poison Control Center of São Paulo. São Paulo – SP/Brazil

^d^Pharmacy College, Federal University of Goiás, Goiânia – GO/Brazil

* To whom correspondence should be addressed: Sarmento Leite Street, 245, Zip code: 90050-170, Porto Alegre - RS, Brazil. Phone: +55 11 95755 8318. E-mail address: sarahcarobini@hotmail.com

Supplementary material S1. Descriptive analysis of frequency of categorical variables within the evaluation variable, classified as less severe (non-toxic, probably non-toxic, intoxication not excluded and mild intoxication) and more severe (moderate to severe).

| **Gender** | **More severe** | **Less severe** | **Total** |
| --- | --- | --- | --- |
| Female | 22 | **92** | 114 |
| Male | 43 | **163** | 206 |
| **Occurrence** | **More severe** | **Less severe** | **Total** |
| Differential diagnosis | 3 | **37** | 40 |
| Exposure | 1 | **21** | 22 |
| Intoxication | 61 | **187** | 248 |
| Other | 0 | **9** | 9 |
| Adverse reaction | 0 | **1** | 1 |
| **Circumstance** | **More severe** | **Less severe** | **Total** |
| Abuse | 35 | **165** | 200 |
| Abuse_suicide attempet | 1 | **3** | 4 |
| Abuse_Violence / Homicide | 0 | **1** | 1 |
| Collective accident | **1** | 0 | 1 |
| Individual Accident | 0 | **8** | 8 |
| Individual Accident _Abuse | 0 | **1** | 1 |
| Individual Accident _ Attempted suicide | 0 | **1** | 1 |
| Self medication | **1** | 0 | 1 |
| Administration error | 0 | **2** | 2 |
| Administration_Abuse error | 0 | **1** | 1 |
| Ignored | 8 | **12** | 20 |
| Another | 1 | 1 | 2 |
| Suicide attempt | 18 | **57** | 75 |
| Therapeutic use | 0 | **1** | 1 |
| Violence / Homicide | 0 | **2** | 2 |
| **Exposure** | **More severe** | **Less severe** | **Total** |
| Cutaneous_Respiratory | 0 | **1** | 1 |
| Ignored | 3 | **14** | 17 |
| Bite / Sting | 0 | **1** | 1 |
| Nasal | 2 | **14** | 16 |
| Oral | 34 | **101** | 135 |
| Oral_Nasal | 4 | **54** | 58 |
| Oral_Parenteral | 0 | **1** | 1 |
| Oral_Respiratory | 9 | **20** | 29 |
| Oral_Respiratória_Nasal | 1 | **9** | 10 |
| Another | 0 | **1** | 1 |
| Respiratory | 11 | **34** | 45 |
| Respiratory_Nasal | 0 | **5** | 5 |
| Respiratory_Parenteral | **1** | 0 | 1 |
| **Type** | **More severe** | **Less severe** | **Total** |
| Acute repeated | 6 | **43** | 49 |
| Acute on chronic | 30 | **54** | 84 |
| Acute single | 10 | **76** | 86 |
| High single_Repeated high | 0 | **1** | 1 |
| Acute single_Acute on chronic | 1 | **3** | 4 |
| Chronicle | 1 | **38** | 39 |
| Ignored | 17 | **40** | 57 |
| **Toxic agent** | **More severe** | **Less severe** | **Total** |
| Pesticides | 1 | **3** | 4 |
| Pesticides_Raticides | 0 | **2** | 2 |
| Foods | 0 | **1** | 1 |
| Drugs of abuse | 34 | **162** | 196 |
| Abuse Drugs | 0 | **1** | 1 |
| Ignored | 3 | **13** | 16 |
| Medicines | 17 | **49** | 66 |
| Medicines_Drugs of abuse | 4 | **12** | 16 |
| Medicines_Veterinary Products_Drugs of Abuse | 0 | **1** | 1 |
| Medicines_Raticides | **2** | 1 | 3 |
| Another | 0 | **4** | 4 |
| Plants | **1** | 0 | 1 |
| Industrial Chemicals | 0 | **1** | 1 |
| Rodenticides | 3 | **3** | 6 |
| Raticidas_Drugs of abuse | 0 | **1** | 1 |
| Household Sanitizers | 0 | **1** | 1 |
| **Evolution** | **More severe** | **Less severe** | **Total** |
| Cure | 12 | **40** | 52 |
| Unconfirmed cure | 3 | **8** | 11 |
| Ignored | 8 | **24** | 32 |
| Death | **2** | 1 | 3 |
| Death another cause | 0 | **1** | 1 |
| Another | 40 | **181** | 221 |

**Supplementary material S2.** Correlation matrix of multiple correspondence analysis.

MULTIPLE CORRESPONDENCE ANALYSIS

ELIMINATION OF ACTIVE CATEGORIES WITH SMALL WEIGHTS

THRESHOLD (PCMIN) : 2.00 % WEIGHT: 6.40

BEFORE CLEANING : 143 ACTIVE QUESTIONS 537 ASSOCIATE CATEGORIES

AFTER CLEANING : 57 ACTIVE QUESTIONS 148 ASSOCIATE CATEGORIES

TOTAL WEIGHT OF ACTIVE CASES : 320.00

MARGINAL DISTRIBUTIONS OF ACTIVE QUESTIONS

MULTIPLE CORRESPONDENCE TABLE

| m1 m3 m4 m5 m6 | m1 m2 | m1 m2 m3 m4 | m1 m5 m11 m13 | m1 m2 | m1 m2 | m1 m2 |

-----+--------------------------+-----------+---------------------+---------------------+-----------+-----------+-----------+

m1 | 45 0 0 0 0 |

m3 | 0 96 0 0 0 |

m4 | 0 0 102 0 0 |

m5 | 0 0 0 56 0 |

m6 | 0 0 0 0 21 |

-----+--------------------------+-----------+

m1 | 22 34 27 22 9 | 114 0 |

m2 | 23 62 75 34 12 | 0 206 |

-----+--------------------------+-----------+---------------------+

m1 | 6 15 9 8 2 | 12 28 | 40 0 0 0 |

m2 | 5 7 7 2 1 | 12 10 | 0 22 0 0 |

m3 | 34 71 81 45 17 | 82 166 | 0 0 248 0 |

m4 | 0 3 5 1 1 | 8 2 | 0 0 0 10 |

-----+--------------------------+-----------+---------------------+---------------------+

m1 | 25 63 75 31 8 | 49 153 | 22 7 166 7 | 202 0 0 0 |

m5 | 0 4 2 2 3 | 6 5 | 2 1 8 0 | 0 11 0 0 |

m11 | 3 6 8 6 2 | 8 17 | 12 2 10 1 | 0 0 25 0 |

m13 | 17 23 17 17 8 | 51 31 | 4 12 64 2 | 0 0 0 82 |

-----+--------------------------+-----------+---------------------+---------------------+-----------+

m1 | 45 90 101 55 19 | 110 200 | 38 21 241 10 | 202 3 24 81 | 310 0 |

m2 | 0 6 1 1 2 | 4 6 | 2 1 7 0 | 0 8 1 1 | 0 10 |

-----+--------------------------+-----------+---------------------+---------------------+-----------+-----------+

m1 | 18 33 26 24 12 | 64 49 | 19 15 76 3 | 2 10 23 78 | 104 9 | 113 0 |

m2 | 27 63 76 32 9 | 50 157 | 21 7 172 7 | 200 1 2 4 | 206 1 | 0 207 |

-----+--------------------------+-----------+---------------------+---------------------+-----------+-----------+-----------+

m1 | 29 74 86 38 13 | 63 177 | 38 11 183 8 | 202 10 22 6 | 231 9 | 37 203 | 240 0 |

m2 | 16 22 16 18 8 | 51 29 | 2 11 65 2 | 0 1 3 76 | 79 1 | 76 4 | 0 80 |

-----+--------------------------+-----------+---------------------+---------------------+-----------+-----------+-----------+

m1 | 42 92 96 51 19 | 107 193 | 29 21 241 9 | 202 11 5 82 | 290 10 | 93 207 | 220 80 |

m2 | 3 4 6 5 2 | 7 13 | 11 1 7 1 | 0 0 20 0 | 20 0 | 20 0 | 20 0 |

-----+--------------------------+-----------+---------------------+---------------------+-----------+-----------+-----------+

m2 | 5 5 6 2 1 | 7 12 | 14 0 3 2 | 7 0 10 2 | 19 0 | 12 7 | 17 2 |

m4 | 1 5 8 2 0 | 6 10 | 1 3 11 1 | 16 0 0 0 | 16 0 | 0 16 | 16 0 |

m5 | 28 36 34 24 14 | 73 63 | 12 16 103 5 | 44 8 9 75 | 130 6 | 92 44 | 63 73 |

m6 | 7 21 19 10 2 | 11 48 | 2 1 56 0 | 52 0 4 3 | 59 0 | 4 55 | 55 4 |

m8 | 1 10 16 4 2 | 3 30 | 5 1 26 1 | 32 0 1 0 | 33 0 | 1 32 | 33 0 |

m9 | 2 2 4 2 1 | 2 9 | 1 0 10 0 | 9 1 0 1 | 10 1 | 1 10 | 10 1 |

m11 | 1 17 15 12 1 | 12 34 | 5 1 39 1 | 42 2 1 1 | 43 3 | 3 43 | 46 0 |

-----+--------------------------+-----------+---------------------+---------------------+-----------+-----------+-----------+

m1 | 8 29 31 16 3 | 29 58 | 20 5 57 5 | 70 2 12 3 | 84 3 | 16 71 | 85 2 |

m2 | 37 67 71 40 18 | 85 148 | 20 17 191 5 | 132 9 13 79 | 226 7 | 97 136 | 155 78 |

-----+--------------------------+-----------+---------------------+---------------------+-----------+-----------+-----------+

m1 | 39 67 67 38 18 | 96 133 | 29 20 171 9 | 119 8 22 80 | 223 6 | 107 122 | 150 79 |

m2 | 6 29 35 18 3 | 18 73 | 11 2 77 1 | 83 3 3 2 | 87 4 | 6 85 | 90 1 |

-----+--------------------------+-----------+---------------------+---------------------+-----------+-----------+-----------+

m1 | 34 68 70 42 18 | 95 137 | 36 18 169 9 | 123 10 21 78 | 223 9 | 108 124 | 157 75 |

m2 | 11 28 32 14 3 | 19 69 | 4 4 79 1 | 79 1 4 4 | 87 1 | 5 83 | 83 5 |

-----+--------------------------+-----------+---------------------+---------------------+-----------+-----------+-----------+

m1 | 41 92 96 54 20 | 109 194 | 26 22 246 9 | 197 11 15 80 | 293 10 | 101 202 | 225 78 |

m2 | 4 4 6 2 1 | 5 12 | 14 0 2 1 | 5 0 10 2 | 17 0 | 12 5 | 15 2 |

-----+--------------------------+-----------+---------------------+---------------------+-----------+-----------+-----------+

m2 | 6 16 18 11 0 | 10 41 | 2 1 48 0 | 48 0 0 3 | 51 0 | 4 47 | 48 3 |

m3 | 5 27 36 11 6 | 26 59 | 8 6 71 0 | 66 3 2 14 | 84 1 | 18 67 | 70 15 |

m4 | 22 28 16 11 9 | 49 37 | 8 12 63 3 | 24 8 4 50 | 77 9 | 59 27 | 37 49 |

m7 | 2 12 15 9 3 | 15 26 | 2 2 33 4 | 35 0 2 4 | 41 0 | 6 35 | 38 3 |

m8 | 10 13 17 14 3 | 14 43 | 20 1 33 3 | 29 0 17 11 | 57 0 | 26 31 | 47 10 |

-----+--------------------------+-----------+---------------------+---------------------+-----------+-----------+-----------+

m4 | 22 66 75 30 8 | 48 153 | 19 8 168 6 | 188 3 3 7 | 197 4 | 11 190 | 197 4 |

m6 | 4 5 5 6 3 | 9 14 | 13 0 8 2 | 3 2 11 7 | 21 2 | 20 3 | 16 7 |

m7 | 16 23 11 13 7 | 46 24 | 6 12 52 0 | 5 6 7 52 | 66 4 | 65 5 | 18 52 |

m8 | 3 2 11 7 3 | 11 15 | 2 2 20 2 | 6 0 4 16 | 26 0 | 17 9 | 9 17 |

-----+--------------------------+-----------+---------------------+---------------------+-----------+-----------+-----------+

m1 | 27 73 81 37 16 | 63 171 | 33 9 184 8 | 193 7 14 20 | 227 7 | 38 196 | 216 18 |

m2 | 18 23 21 19 5 | 51 35 | 7 13 64 2 | 9 4 11 62 | 83 3 | 75 11 | 24 62 |

-----+--------------------------+-----------+---------------------+---------------------+-----------+-----------+-----------+

m1 | 44 92 99 54 18 | 105 202 | 40 21 237 9 | 202 10 25 70 | 297 10 | 102 205 | 239 68 |

m2 | 1 4 3 2 3 | 9 4 | 0 1 11 1 | 0 1 0 12 | 13 0 | 11 2 | 1 12 |

-----+--------------------------+-----------+---------------------+---------------------+-----------+-----------+-----------+

m1 | 21 31 18 22 13 | 61 44 | 20 14 68 3 | 10 9 18 68 | 97 8 | 96 9 | 38 67 |

m2 | 24 65 84 34 8 | 53 162 | 20 8 180 7 | 192 2 7 14 | 213 2 | 17 198 | 202 13 |

-----+--------------------------+-----------+---------------------+---------------------+-----------+-----------+-----------+

m1 | 42 93 98 52 19 | 110 194 | 27 22 246 9 | 199 9 15 81 | 296 8 | 100 204 | 225 79 |

m2 | 3 3 4 4 2 | 4 12 | 13 0 2 1 | 3 2 10 1 | 14 2 | 13 3 | 15 1 |

-----+--------------------------+-----------+---------------------+---------------------+-----------+-----------+-----------+

| m1 m3 m4 m5 m6 | m1 m2 | m1 m2 m3 m4 | m1 m5 m11 m13 | m1 m2 | m1 m2 | m1 m2 |

| m1 m3 m4 m5 m6 | m1 m2 | m1 m2 m3 m4 | m1 m5 m11 m13 | m1 m2 | m1 m2 | m1 m2 |

-----+--------------------------+-----------+---------------------+---------------------+-----------+-----------+-----------+

m1 | 3 16 21 9 5 | 23 31 | 6 5 40 3 | 31 5 1 17 | 49 5 | 23 31 | 39 15 |

m2 | 0 5 4 3 0 | 5 7 | 2 1 9 0 | 10 0 1 1 | 12 0 | 2 10 | 11 1 |

m3 | 38 66 69 37 12 | 74 148 | 27 14 176 5 | 146 4 19 53 | 218 4 | 75 147 | 170 52 |

m6 | 4 9 8 7 4 | 12 20 | 5 2 23 2 | 15 2 4 11 | 31 1 | 13 19 | 20 12 |

-----+--------------------------+-----------+---------------------+---------------------+-----------+-----------+-----------+

m1 | 14 21 21 13 3 | 26 46 | 7 4 58 3 | 43 2 7 20 | 69 3 | 28 44 | 51 21 |

m8 | 1 10 6 0 2 | 9 10 | 3 5 10 1 | 10 0 1 8 | 19 0 | 9 10 | 11 8 |

m11 | 10 22 19 10 2 | 27 36 | 11 2 46 4 | 41 4 3 15 | 60 3 | 23 40 | 50 13 |

m14 | 15 32 47 25 12 | 38 93 | 16 9 104 2 | 87 2 12 30 | 129 2 | 41 90 | 104 27 |

m26 | 1 9 7 7 1 | 9 16 | 1 2 22 0 | 14 3 1 7 | 23 2 | 9 16 | 16 9 |

m31 | 4 2 2 1 1 | 5 5 | 2 0 8 0 | 7 0 1 2 | 10 0 | 3 7 | 8 2 |

-----+--------------------------+-----------+---------------------+---------------------+-----------+-----------+-----------+

m1 | 19 29 33 22 5 | 39 69 | 9 5 90 4 | 68 4 9 27 | 103 5 | 38 70 | 79 29 |

m2 | 26 67 69 34 16 | 75 137 | 31 17 158 6 | 134 7 16 55 | 207 5 | 75 137 | 161 51 |

-----+--------------------------+-----------+---------------------+---------------------+-----------+-----------+-----------+

m1 | 16 28 28 17 4 | 37 56 | 12 5 72 4 | 60 2 9 22 | 90 3 | 32 61 | 70 23 |

m2 | 29 68 74 39 17 | 77 150 | 28 17 176 6 | 142 9 16 60 | 220 7 | 81 146 | 170 57 |

-----+--------------------------+-----------+---------------------+---------------------+-----------+-----------+-----------+

m1 | 29 48 46 21 8 | 57 95 | 23 11 112 6 | 100 8 12 32 | 145 7 | 51 101 | 120 32 |

m2 | 16 48 56 35 13 | 57 111 | 17 11 136 4 | 102 3 13 50 | 165 3 | 62 106 | 120 48 |

-----+--------------------------+-----------+---------------------+---------------------+-----------+-----------+-----------+

m1 | 40 90 99 51 18 | 97 201 | 40 13 235 10 | 202 10 25 61 | 288 10 | 92 206 | 240 58 |

m2 | 5 6 3 5 3 | 17 5 | 0 9 13 0 | 0 1 0 21 | 22 0 | 21 1 | 0 22 |

-----+--------------------------+-----------+---------------------+---------------------+-----------+-----------+-----------+

m1 | 42 92 100 53 18 | 103 202 | 40 16 239 10 | 202 10 25 68 | 295 10 | 99 206 | 240 65 |

m2 | 3 4 2 3 3 | 11 4 | 0 6 9 0 | 0 1 0 14 | 15 0 | 14 1 | 0 15 |

-----+--------------------------+-----------+---------------------+---------------------+-----------+-----------+-----------+

m1 | 34 76 81 43 18 | 90 162 | 33 20 192 7 | 160 9 18 65 | 245 7 | 88 164 | 190 62 |

m2 | 11 20 21 13 3 | 24 44 | 7 2 56 3 | 42 2 7 17 | 65 3 | 25 43 | 50 18 |

-----+--------------------------+-----------+---------------------+---------------------+-----------+-----------+-----------+

m1 | 1 2 2 6 1 | 7 5 | 0 1 11 0 | 4 0 1 7 | 12 0 | 8 4 | 6 6 |

m2 | 24 63 62 31 11 | 63 128 | 12 4 171 4 | 138 5 8 40 | 185 6 | 48 143 | 150 41 |

m3 | 5 13 22 11 6 | 17 40 | 4 1 52 0 | 32 2 9 14 | 57 0 | 25 32 | 42 15 |

m4 | 14 16 12 5 3 | 24 26 | 21 14 11 4 | 20 4 5 21 | 46 4 | 30 20 | 32 18 |

m5 | 1 2 4 3 0 | 3 7 | 3 2 3 2 | 8 0 2 0 | 10 0 | 2 8 | 10 0 |

-----+--------------------------+-----------+---------------------+---------------------+-----------+-----------+-----------+

m1 | 38 76 79 46 18 | 97 160 | 33 19 195 10 | 159 10 20 68 | 248 9 | 95 162 | 191 66 |

m5 | 4 12 14 5 2 | 12 25 | 3 3 31 0 | 22 1 4 10 | 36 1 | 13 24 | 27 10 |

m26 | 3 8 9 5 1 | 5 21 | 4 0 22 0 | 21 0 1 4 | 26 0 | 5 21 | 22 4 |

-----+--------------------------+-----------+---------------------+---------------------+-----------+-----------+-----------+

m1 | 43 83 91 53 19 | 107 182 | 40 22 217 10 | 173 10 25 81 | 279 10 | 113 176 | 210 79 |

m2 | 2 13 11 3 2 | 7 24 | 0 0 31 0 | 29 1 0 1 | 31 0 | 0 31 | 30 1 |

-----+--------------------------+-----------+---------------------+---------------------+-----------+-----------+-----------+

m1 | 44 88 98 55 20 | 112 193 | 40 22 233 10 | 191 11 23 80 | 296 9 | 109 196 | 228 77 |

m2 | 1 8 4 1 1 | 2 13 | 0 0 15 0 | 11 0 2 2 | 14 1 | 4 11 | 12 3 |

-----+--------------------------+-----------+---------------------+---------------------+-----------+-----------+-----------+

m1 | 41 94 95 55 21 | 109 197 | 38 19 239 10 | 195 11 23 77 | 296 10 | 107 199 | 230 76 |

m2 | 4 2 7 1 0 | 5 9 | 2 3 9 0 | 7 0 2 5 | 14 0 | 6 8 | 10 4 |

-----+--------------------------+-----------+---------------------+---------------------+-----------+-----------+-----------+

m1 | 45 94 99 50 21 | 109 200 | 39 22 238 10 | 195 11 24 79 | 299 10 | 110 199 | 233 76 |

m2 | 0 2 3 6 0 | 5 6 | 1 0 10 0 | 7 0 1 3 | 11 0 | 3 8 | 7 4 |

-----+--------------------------+-----------+---------------------+---------------------+-----------+-----------+-----------+

m1 | 23 57 50 23 10 | 59 104 | 17 21 120 5 | 113 5 9 36 | 156 7 | 48 115 | 128 35 |

m4 | 7 8 15 7 0 | 13 24 | 2 0 34 1 | 24 2 1 10 | 36 1 | 13 24 | 27 10 |

m5 | 2 7 10 3 2 | 8 16 | 1 0 22 1 | 16 0 2 6 | 24 0 | 7 17 | 18 6 |

m52 | 3 7 8 4 6 | 11 17 | 7 0 20 1 | 14 1 4 9 | 28 0 | 13 15 | 20 8 |

m54 | 1 5 6 6 2 | 11 9 | 4 0 16 0 | 4 2 5 9 | 19 1 | 16 4 | 11 9 |

m57 | 5 6 8 3 1 | 3 20 | 4 1 16 2 | 18 0 2 3 | 23 0 | 5 18 | 20 3 |

m60 | 4 6 5 10 0 | 9 16 | 5 0 20 0 | 13 1 2 9 | 24 1 | 11 14 | 16 9 |

-----+--------------------------+-----------+---------------------+---------------------+-----------+-----------+-----------+

m1 | 45 93 100 51 21 | 110 200 | 38 22 240 10 | 200 11 23 76 | 300 10 | 105 205 | 236 74 |

m2 | 0 3 2 5 0 | 4 6 | 2 0 8 0 | 2 0 2 6 | 10 0 | 8 2 | 4 6 |

-----+--------------------------+-----------+---------------------+---------------------+-----------+-----------+-----------+

m1 | 43 93 99 53 21 | 108 201 | 35 22 242 10 | 201 11 21 76 | 299 10 | 103 206 | 235 74 |

m2 | 2 3 3 3 0 | 6 5 | 5 0 6 0 | 1 0 4 6 | 11 0 | 10 1 | 5 6 |

-----+--------------------------+-----------+---------------------+---------------------+-----------+-----------+-----------+

m1 | 42 89 94 47 19 | 98 193 | 39 22 220 10 | 194 9 22 66 | 282 9 | 93 198 | 228 63 |

m2 | 3 7 8 9 2 | 16 13 | 1 0 28 0 | 8 2 3 16 | 28 1 | 20 9 | 12 17 |

-----+--------------------------+-----------+---------------------+---------------------+-----------+-----------+-----------+

m1 | 36 79 79 47 20 | 99 162 | 34 22 197 8 | 154 9 21 77 | 252 9 | 102 159 | 186 75 |

m2 | 9 17 23 9 1 | 15 44 | 6 0 51 2 | 48 2 4 5 | 58 1 | 11 48 | 54 5 |

-----+--------------------------+-----------+---------------------+---------------------+-----------+-----------+-----------+

m1 | 41 90 93 54 20 | 108 190 | 37 22 231 8 | 183 11 23 81 | 288 10 | 110 188 | 219 79 |

m2 | 4 6 9 2 1 | 6 16 | 3 0 17 2 | 19 0 2 1 | 22 0 | 3 19 | 21 1 |

-----+--------------------------+-----------+---------------------+---------------------+-----------+-----------+-----------+

| m1 m3 m4 m5 m6 | m1 m2 | m1 m2 m3 m4 | m1 m5 m11 m13 | m1 m2 | m1 m2 | m1 m2 |

| m1 m3 m4 m5 m6 | m1 m2 | m1 m2 m3 m4 | m1 m5 m11 m13 | m1 m2 | m1 m2 | m1 m2 |

-----+--------------------------+-----------+---------------------+---------------------+-----------+-----------+-----------+

m1 | 44 92 96 55 21 | 113 195 | 40 21 237 10 | 192 11 25 80 | 298 10 | 111 197 | 230 78 |

m2 | 1 4 6 1 0 | 1 11 | 0 1 11 0 | 10 0 0 2 | 12 0 | 2 10 | 10 2 |

-----+--------------------------+-----------+---------------------+---------------------+-----------+-----------+-----------+

m1 | 42 92 91 49 16 | 100 190 | 35 22 224 9 | 195 9 20 66 | 280 10 | 92 198 | 225 65 |

m2 | 3 4 11 7 5 | 14 16 | 5 0 24 1 | 7 2 5 16 | 30 0 | 21 9 | 15 15 |

-----+--------------------------+-----------+---------------------+---------------------+-----------+-----------+-----------+

m1 | 45 94 98 54 21 | 112 200 | 40 22 240 10 | 195 11 25 81 | 302 10 | 112 200 | 233 79 |

m2 | 0 2 4 2 0 | 2 6 | 0 0 8 0 | 7 0 0 1 | 8 0 | 1 7 | 7 1 |

-----+--------------------------+-----------+---------------------+---------------------+-----------+-----------+-----------+

m1 | 45 93 99 54 18 | 112 197 | 37 22 240 10 | 193 11 23 82 | 300 9 | 111 198 | 230 79 |

m2 | 0 3 3 2 3 | 2 9 | 3 0 8 0 | 9 0 2 0 | 10 1 | 2 9 | 10 1 |

-----+--------------------------+-----------+---------------------+---------------------+-----------+-----------+-----------+

m1 | 38 78 88 47 15 | 96 170 | 37 19 200 10 | 171 10 21 64 | 258 8 | 93 173 | 202 64 |

m4 | 6 2 5 2 1 | 4 12 | 1 1 14 0 | 14 0 0 2 | 16 0 | 2 14 | 14 2 |

m12 | 1 16 9 7 5 | 14 24 | 2 2 34 0 | 17 1 4 16 | 36 2 | 18 20 | 24 14 |

-----+--------------------------+-----------+---------------------+---------------------+-----------+-----------+-----------+

m1 | 42 79 92 49 15 | 97 180 | 38 20 209 10 | 182 10 22 63 | 269 8 | 93 184 | 214 63 |

m2 | 3 17 10 7 6 | 17 26 | 2 2 39 0 | 20 1 3 19 | 41 2 | 20 23 | 26 17 |

-----+--------------------------+-----------+---------------------+---------------------+-----------+-----------+-----------+

m1 | 39 93 98 52 19 | 108 193 | 39 22 230 10 | 188 10 25 78 | 291 10 | 110 191 | 225 76 |

m2 | 6 3 4 4 2 | 6 13 | 1 0 18 0 | 14 1 0 4 | 19 0 | 3 16 | 15 4 |

-----+--------------------------+-----------+---------------------+---------------------+-----------+-----------+-----------+

m1 | 44 91 99 55 20 | 108 201 | 40 21 238 10 | 197 11 25 76 | 299 10 | 107 202 | 235 74 |

m2 | 1 5 3 1 1 | 6 5 | 0 1 10 0 | 5 0 0 6 | 11 0 | 6 5 | 5 6 |

-----+--------------------------+-----------+---------------------+---------------------+-----------+-----------+-----------+

m1 | 45 87 97 44 19 | 105 187 | 38 22 222 10 | 182 11 22 77 | 282 10 | 106 186 | 218 74 |

m4 | 0 9 5 12 2 | 9 19 | 2 0 26 0 | 20 0 3 5 | 28 0 | 7 21 | 22 6 |

-----+--------------------------+-----------+---------------------+---------------------+-----------+-----------+-----------+

m1 | 45 94 102 51 21 | 110 203 | 38 22 243 10 | 200 11 23 79 | 303 10 | 108 205 | 236 77 |

m2 | 0 2 0 5 0 | 4 3 | 2 0 5 0 | 2 0 2 3 | 7 0 | 5 2 | 4 3 |

-----+--------------------------+-----------+---------------------+---------------------+-----------+-----------+-----------+

m1 | 45 93 102 52 21 | 110 203 | 40 22 241 10 | 198 9 24 82 | 305 8 | 111 202 | 234 79 |

m2 | 0 3 0 4 0 | 4 3 | 0 0 7 0 | 4 2 1 0 | 5 2 | 2 5 | 6 1 |

-----+--------------------------+-----------+---------------------+---------------------+-----------+-----------+-----------+

m1 | 45 87 99 52 20 | 106 197 | 39 22 232 10 | 190 10 24 79 | 294 9 | 108 195 | 226 77 |

m2 | 0 9 3 4 1 | 8 9 | 1 0 16 0 | 12 1 1 3 | 16 1 | 5 12 | 14 3 |

-----+--------------------------+-----------+---------------------+---------------------+-----------+-----------+-----------+

m1 | 45 95 101 53 18 | 109 203 | 40 22 240 10 | 200 11 23 78 | 302 10 | 108 204 | 237 75 |

m2 | 0 1 1 3 3 | 5 3 | 0 0 8 0 | 2 0 2 4 | 8 0 | 5 3 | 3 5 |

-----+--------------------------+-----------+---------------------+---------------------+-----------+-----------+-----------+

m1 | 40 70 77 45 17 | 97 152 | 32 20 187 10 | 146 11 20 72 | 240 9 | 101 148 | 179 70 |

m7 | 2 16 17 6 1 | 9 33 | 4 1 37 0 | 36 0 0 6 | 41 1 | 5 37 | 38 4 |

m23 | 3 10 8 5 3 | 8 21 | 4 1 24 0 | 20 0 5 4 | 29 0 | 7 22 | 23 6 |

-----+--------------------------+-----------+---------------------+---------------------+-----------+-----------+-----------+

m1 | 42 84 88 50 21 | 107 178 | 37 21 217 10 | 169 11 25 80 | 276 9 | 112 173 | 206 79 |

m2 | 3 12 14 6 0 | 7 28 | 3 1 31 0 | 33 0 0 2 | 34 1 | 1 34 | 34 1 |

-----+--------------------------+-----------+---------------------+---------------------+-----------+-----------+-----------+

m1 | 44 94 100 54 20 | 113 199 | 40 21 241 10 | 196 11 24 81 | 303 9 | 112 200 | 232 80 |

m2 | 1 2 2 2 1 | 1 7 | 0 1 7 0 | 6 0 1 1 | 7 1 | 1 7 | 8 0 |

-----+--------------------------+-----------+---------------------+---------------------+-----------+-----------+-----------+

m1 | 39 82 91 49 19 | 104 176 | 36 20 214 10 | 172 11 19 78 | 271 9 | 106 174 | 205 75 |

m2 | 6 14 11 7 2 | 10 30 | 4 2 34 0 | 30 0 6 4 | 39 1 | 7 33 | 35 5 |

-----+--------------------------+-----------+---------------------+---------------------+-----------+-----------+-----------+

m1 | 45 92 99 54 20 | 112 198 | 40 22 238 10 | 194 11 24 81 | 300 10 | 111 199 | 231 79 |

m2 | 0 4 3 2 1 | 2 8 | 0 0 10 0 | 8 0 1 1 | 10 0 | 2 8 | 9 1 |

-----+--------------------------+-----------+---------------------+---------------------+-----------+-----------+-----------+

| m1 m3 m4 m5 m6 | m1 m2 | m1 m2 m3 m4 | m1 m5 m11 m13 | m1 m2 | m1 m2 | m1 m2 |

| m1 m2 | m2 m4 m5 m6 m8 m9 m11 | m1 m2 | m1 m2 | m1 m2 | m1 m2 | m2 m3 m4 m7 m8 |

-----+-----------+------------------------------------+-----------+-----------+-----------+-----------+--------------------------+

m1 | 300 0 |

m2 | 0 20 |

-----+-----------+------------------------------------+

m2 | 10 9 | 19 0 0 0 0 0 0 |

m4 | 16 0 | 0 16 0 0 0 0 0 |

m5 | 129 7 | 0 0 136 0 0 0 0 |

m6 | 57 2 | 0 0 0 59 0 0 0 |

m8 | 32 1 | 0 0 0 0 33 0 0 |

m9 | 11 0 | 0 0 0 0 0 11 0 |

m11 | 45 1 | 0 0 0 0 0 0 46 |

-----+-----------+------------------------------------+-----------+

m1 | 76 11 | 19 16 1 1 4 1 45 | 87 0 |

m2 | 224 9 | 0 0 135 58 29 10 1 | 0 233 |

-----+-----------+------------------------------------+-----------+-----------+

m1 | 212 17 | 18 16 135 58 1 0 1 | 35 194 | 229 0 |

m2 | 88 3 | 1 0 1 1 32 11 45 | 52 39 | 0 91 |

-----+-----------+------------------------------------+-----------+-----------+-----------+

| m1 m2 | m2 m4 m5 m6 m8 m9 m11 | m1 m2 | m1 m2 | m1 m2 | m1 m2 | m2 m3 m4 m7 m8 |

| m1 m2 | m2 m4 m5 m6 m8 m9 m11 | m1 m2 | m1 m2 | m1 m2 | m1 m2 | m2 m3 m4 m7 m8 |

-----+-----------+------------------------------------+-----------+-----------+-----------+-----------+--------------------------+

m1 | 214 18 | 18 0 136 1 31 0 46 | 67 165 | 155 77 | 232 0 |

m2 | 86 2 | 1 16 0 58 2 11 0 | 20 68 | 74 14 | 0 88 |

-----+-----------+------------------------------------+-----------+-----------+-----------+-----------+

m1 | 292 11 | 2 16 136 59 33 11 46 | 70 233 | 212 91 | 215 88 | 303 0 |

m2 | 8 9 | 17 0 0 0 0 0 0 | 17 0 | 17 0 | 17 0 | 0 17 |

-----+-----------+------------------------------------+-----------+-----------+-----------+-----------+--------------------------+

m2 | 51 0 | 0 1 9 7 10 2 22 | 25 26 | 16 35 | 41 10 | 51 0 | 51 0 0 0 0 |

m3 | 83 2 | 2 8 24 19 11 8 13 | 24 61 | 53 32 | 48 37 | 84 1 | 0 85 0 0 0 |

m4 | 85 1 | 0 3 67 7 2 0 7 | 10 76 | 77 9 | 76 10 | 86 0 | 0 0 86 0 0 |

m7 | 39 2 | 0 2 14 19 3 0 3 | 6 35 | 35 6 | 20 21 | 41 0 | 0 0 0 41 0 |

m8 | 42 15 | 17 2 22 7 7 1 1 | 22 35 | 48 9 | 47 10 | 41 16 | 0 0 0 0 57 |

-----+-----------+------------------------------------+-----------+-----------+-----------+-----------+--------------------------+

m4 | 199 2 | 6 16 41 55 30 10 43 | 69 132 | 117 84 | 118 83 | 197 4 | 47 62 28 36 28 |

m6 | 13 10 | 10 0 11 0 1 0 1 | 11 12 | 21 2 | 23 0 | 13 10 | 0 1 9 0 13 |

m7 | 64 6 | 3 0 65 0 1 0 1 | 4 66 | 68 2 | 70 0 | 67 3 | 2 15 37 4 12 |

m8 | 24 2 | 0 0 19 4 1 1 1 | 3 23 | 23 3 | 21 5 | 26 0 | 2 7 12 1 4 |

-----+-----------+------------------------------------+-----------+-----------+-----------+-----------+--------------------------+

m1 | 222 12 | 16 16 59 55 32 10 46 | 83 151 | 146 88 | 151 83 | 220 14 | 47 65 44 36 42 |

m2 | 78 8 | 3 0 77 4 1 1 0 | 4 82 | 83 3 | 81 5 | 83 3 | 4 20 42 5 15 |

-----+-----------+------------------------------------+-----------+-----------+-----------+-----------+--------------------------+

m1 | 287 20 | 19 16 123 59 33 11 46 | 87 220 | 216 91 | 219 88 | 290 17 | 51 84 74 41 57 |

m2 | 13 0 | 0 0 13 0 0 0 0 | 0 13 | 13 0 | 13 0 | 13 0 | 0 1 12 0 0 |

-----+-----------+------------------------------------+-----------+-----------+-----------+-----------+--------------------------+

m1 | 89 16 | 13 0 85 0 3 0 4 | 18 87 | 99 6 | 105 0 | 92 13 | 2 16 57 4 26 |

m2 | 211 4 | 6 16 51 59 30 11 42 | 69 146 | 130 85 | 127 88 | 211 4 | 49 69 29 37 31 |

-----+-----------+------------------------------------+-----------+-----------+-----------+-----------+--------------------------+

m1 | 293 11 | 10 16 131 59 32 11 45 | 77 227 | 215 89 | 216 88 | 296 8 | 51 84 83 41 45 |

m2 | 7 9 | 9 0 5 0 1 0 1 | 10 6 | 14 2 | 16 0 | 7 9 | 0 1 3 0 12 |

-----+-----------+------------------------------------+-----------+-----------+-----------+-----------+--------------------------+

m1 | 53 1 | 1 4 27 6 6 1 9 | 15 39 | 38 16 | 43 11 | 54 0 | 11 13 16 10 4 |

m2 | 11 1 | 1 3 2 4 1 0 1 | 5 7 | 10 2 | 5 7 | 11 1 | 1 8 0 1 2 |

m3 | 207 15 | 13 8 94 44 23 8 32 | 57 165 | 158 64 | 161 61 | 209 13 | 35 51 64 29 43 |

m6 | 29 3 | 4 1 13 5 3 2 4 | 10 22 | 23 9 | 23 9 | 29 3 | 4 13 6 1 8 |

-----+-----------+------------------------------------+-----------+-----------+-----------+-----------+--------------------------+

m1 | 68 4 | 3 3 33 15 7 3 8 | 15 57 | 55 17 | 51 21 | 69 3 | 16 7 18 14 17 |

m8 | 19 0 | 1 0 11 2 2 0 3 | 6 13 | 14 5 | 16 3 | 18 1 | 2 3 8 5 1 |

m11 | 60 3 | 6 4 28 14 4 0 7 | 16 47 | 52 11 | 44 19 | 58 5 | 6 20 19 7 11 |

m14 | 119 12 | 8 7 49 23 16 7 21 | 39 92 | 86 45 | 93 38 | 124 7 | 22 43 30 13 23 |

m26 | 25 0 | 0 2 10 4 3 1 5 | 8 17 | 15 10 | 19 6 | 25 0 | 4 10 8 1 2 |

m31 | 9 1 | 1 0 5 1 1 0 2 | 3 7 | 7 3 | 9 1 | 9 1 | 1 2 3 1 3 |

-----+-----------+------------------------------------+-----------+-----------+-----------+-----------+--------------------------+

m1 | 103 5 | 4 5 45 21 11 3 19 | 30 78 | 75 33 | 80 28 | 104 4 | 23 16 28 19 22 |

m2 | 197 15 | 15 11 91 38 22 8 27 | 57 155 | 154 58 | 152 60 | 199 13 | 28 69 58 22 35 |

-----+-----------+------------------------------------+-----------+-----------+-----------+-----------+--------------------------+

m1 | 88 5 | 5 3 41 19 9 3 13 | 24 69 | 69 24 | 67 26 | 88 5 | 19 10 23 21 20 |

m2 | 212 15 | 14 13 95 40 24 8 33 | 63 164 | 160 67 | 165 62 | 215 12 | 32 75 63 20 37 |

-----+-----------+------------------------------------+-----------+-----------+-----------+-----------+--------------------------+

m1 | 144 8 | 11 7 64 34 14 3 19 | 39 113 | 117 35 | 106 46 | 142 10 | 24 29 42 27 30 |

m2 | 156 12 | 8 9 72 25 19 8 27 | 48 120 | 112 56 | 126 42 | 161 7 | 27 56 44 14 27 |

-----+-----------+------------------------------------+-----------+-----------+-----------+-----------+--------------------------+

m1 | 278 20 | 19 16 114 59 33 11 46 | 87 211 | 207 91 | 210 88 | 281 17 | 51 78 73 41 55 |

m2 | 22 0 | 0 0 22 0 0 0 0 | 0 22 | 22 0 | 22 0 | 22 0 | 0 7 13 0 2 |

-----+-----------+------------------------------------+-----------+-----------+-----------+-----------+--------------------------+

m1 | 285 20 | 19 16 121 59 33 11 46 | 87 218 | 214 91 | 217 88 | 288 17 | 51 82 75 41 56 |

m2 | 15 0 | 0 0 15 0 0 0 0 | 0 15 | 15 0 | 15 0 | 15 0 | 0 3 11 0 1 |

-----+-----------+------------------------------------+-----------+-----------+-----------+-----------+--------------------------+

m1 | 236 16 | 16 13 106 45 26 8 38 | 72 180 | 178 74 | 184 68 | 238 14 | 35 79 70 28 40 |

m2 | 64 4 | 3 3 30 14 7 3 8 | 15 53 | 51 17 | 48 20 | 65 3 | 16 6 16 13 17 |

-----+-----------+------------------------------------+-----------+-----------+-----------+-----------+--------------------------+

m1 | 11 1 | 0 0 9 0 0 0 3 | 4 8 | 8 4 | 12 0 | 12 0 | 3 5 3 0 1 |

m2 | 186 5 | 4 12 66 50 22 10 27 | 47 144 | 132 59 | 117 74 | 188 3 | 40 47 50 31 23 |

m3 | 49 8 | 4 2 29 4 9 1 8 | 14 43 | 39 18 | 50 7 | 53 4 | 3 25 10 2 17 |

m4 | 45 5 | 9 1 31 3 1 0 5 | 15 35 | 44 6 | 46 4 | 41 9 | 3 8 21 5 13 |

m5 | 9 1 | 2 1 1 2 1 0 3 | 7 3 | 6 4 | 7 3 | 9 1 | 2 0 2 3 3 |

-----+-----------+------------------------------------+-----------+-----------+-----------+-----------+--------------------------+

m1 | 241 16 | 16 12 113 50 27 8 31 | 64 193 | 192 65 | 186 71 | 242 15 | 37 64 69 38 49 |

m5 | 34 3 | 2 1 15 5 4 1 9 | 13 24 | 21 16 | 29 8 | 36 1 | 6 13 13 1 4 |

m26 | 25 1 | 1 3 8 4 2 2 6 | 10 16 | 16 10 | 17 9 | 25 1 | 8 8 4 2 4 |

-----+-----------+------------------------------------+-----------+-----------+-----------+-----------+--------------------------+

m1 | 269 20 | 19 13 129 54 27 10 37 | 75 214 | 214 75 | 210 79 | 272 17 | 41 71 83 39 55 |

m2 | 31 0 | 0 3 7 5 6 1 9 | 12 19 | 15 16 | 22 9 | 31 0 | 10 14 3 2 2 |

-----+-----------+------------------------------------+-----------+-----------+-----------+-----------+--------------------------+

m1 | 286 19 | 18 15 131 57 30 11 43 | 82 223 | 220 85 | 220 85 | 289 16 | 47 78 83 41 56 |

m2 | 14 1 | 1 1 5 2 3 0 3 | 5 10 | 9 6 | 12 3 | 14 1 | 4 7 3 0 1 |

-----+-----------+------------------------------------+-----------+-----------+-----------+-----------+--------------------------+

| m1 m2 | m2 m4 m5 m6 m8 m9 m11 | m1 m2 | m1 m2 | m1 m2 | m1 m2 | m2 m3 m4 m7 m8 |

| m1 m2 | m2 m4 m5 m6 m8 m9 m11 | m1 m2 | m1 m2 | m1 m2 | m1 m2 | m2 m3 m4 m7 m8 |

-----+-----------+------------------------------------+-----------+-----------+-----------+-----------+--------------------------+

m1 | 288 18 | 18 16 129 56 33 10 44 | 83 223 | 220 86 | 223 83 | 289 17 | 49 80 81 41 55 |

m2 | 12 2 | 1 0 7 3 0 1 2 | 4 10 | 9 5 | 9 5 | 14 0 | 2 5 5 0 2 |

-----+-----------+------------------------------------+-----------+-----------+-----------+-----------+--------------------------+

m1 | 289 20 | 19 16 133 58 33 10 40 | 81 228 | 225 84 | 223 86 | 292 17 | 46 83 83 41 56 |

m2 | 11 0 | 0 0 3 1 0 1 6 | 6 5 | 4 7 | 9 2 | 11 0 | 5 2 3 0 1 |

-----+-----------+------------------------------------+-----------+-----------+-----------+-----------+--------------------------+

m1 | 157 6 | 5 11 63 39 13 4 28 | 45 118 | 119 44 | 108 55 | 159 4 | 28 36 48 26 25 |

m4 | 36 1 | 1 1 13 7 6 1 8 | 11 26 | 21 16 | 29 8 | 36 1 | 8 11 12 4 2 |

m5 | 22 2 | 3 0 9 1 5 3 3 | 7 17 | 13 11 | 20 4 | 21 3 | 2 9 2 4 7 |

m52 | 24 4 | 4 2 15 2 2 0 3 | 9 19 | 22 6 | 23 5 | 25 3 | 3 7 8 1 9 |

m54 | 16 4 | 3 0 14 0 1 1 1 | 4 16 | 17 3 | 19 1 | 17 3 | 1 9 5 0 5 |

m57 | 21 2 | 3 2 7 7 1 1 2 | 8 15 | 20 3 | 13 10 | 20 3 | 2 9 3 3 6 |

m60 | 24 1 | 0 0 15 3 5 1 1 | 3 22 | 17 8 | 20 5 | 25 0 | 7 4 8 3 3 |

-----+-----------+------------------------------------+-----------+-----------+-----------+-----------+--------------------------+

m1 | 292 18 | 18 16 128 59 32 11 46 | 86 224 | 220 90 | 222 88 | 294 16 | 50 80 85 41 54 |

m2 | 8 2 | 1 0 8 0 1 0 0 | 1 9 | 9 1 | 10 0 | 9 1 | 1 5 1 0 3 |

-----+-----------+------------------------------------+-----------+-----------+-----------+-----------+--------------------------+

m1 | 293 16 | 14 16 131 59 33 11 45 | 81 228 | 219 90 | 221 88 | 297 12 | 50 83 83 41 52 |

m2 | 7 4 | 5 0 5 0 0 0 1 | 6 5 | 10 1 | 11 0 | 6 5 | 1 2 3 0 5 |

-----+-----------+------------------------------------+-----------+-----------+-----------+-----------+--------------------------+

m1 | 273 18 | 19 16 112 56 33 10 45 | 86 205 | 202 89 | 207 84 | 274 17 | 47 77 74 39 54 |

m2 | 27 2 | 0 0 24 3 0 1 1 | 1 28 | 27 2 | 25 4 | 29 0 | 4 8 12 2 3 |

-----+-----------+------------------------------------+-----------+-----------+-----------+-----------+--------------------------+

m1 | 245 16 | 15 13 118 50 24 7 34 | 66 195 | 196 65 | 188 73 | 248 13 | 42 68 74 31 46 |

m2 | 55 4 | 4 3 18 9 9 4 12 | 21 38 | 33 26 | 44 15 | 55 4 | 9 17 12 10 11 |

-----+-----------+------------------------------------+-----------+-----------+-----------+-----------+--------------------------+

m1 | 280 18 | 17 15 127 57 31 7 44 | 81 217 | 215 83 | 217 81 | 283 15 | 50 77 86 35 50 |

m2 | 20 2 | 2 1 9 2 2 4 2 | 6 16 | 14 8 | 15 7 | 20 2 | 1 8 0 6 7 |

-----+-----------+------------------------------------+-----------+-----------+-----------+-----------+--------------------------+

m1 | 288 20 | 19 15 134 52 31 11 46 | 86 222 | 219 89 | 228 80 | 291 17 | 50 77 85 40 56 |

m2 | 12 0 | 0 1 2 7 2 0 0 | 1 11 | 10 2 | 4 8 | 12 0 | 1 8 1 1 1 |

-----+-----------+------------------------------------+-----------+-----------+-----------+-----------+--------------------------+

m1 | 275 15 | 15 16 115 58 31 10 45 | 82 208 | 204 86 | 205 85 | 276 14 | 51 76 73 41 49 |

m2 | 25 5 | 4 0 21 1 2 1 1 | 5 25 | 25 5 | 27 3 | 27 3 | 0 9 13 0 8 |

-----+-----------+------------------------------------+-----------+-----------+-----------+-----------+--------------------------+

m1 | 292 20 | 19 15 136 55 30 11 46 | 85 227 | 224 88 | 230 82 | 295 17 | 49 80 86 41 56 |

m2 | 8 0 | 0 1 0 4 3 0 0 | 2 6 | 5 3 | 2 6 | 8 0 | 2 5 0 0 1 |

-----+-----------+------------------------------------+-----------+-----------+-----------+-----------+--------------------------+

m1 | 290 19 | 18 15 131 59 30 10 46 | 85 224 | 222 87 | 223 86 | 293 16 | 50 82 83 40 54 |

m2 | 10 1 | 1 1 5 0 3 1 0 | 2 9 | 7 4 | 9 2 | 10 1 | 1 3 3 1 3 |

-----+-----------+------------------------------------+-----------+-----------+-----------+-----------+--------------------------+

m1 | 249 17 | 16 14 114 47 29 10 36 | 73 193 | 190 76 | 194 72 | 251 15 | 42 70 67 37 50 |

m4 | 16 0 | 1 1 2 4 2 1 5 | 6 10 | 8 8 | 9 7 | 16 0 | 5 6 3 1 1 |

m12 | 35 3 | 2 1 20 8 2 0 5 | 8 30 | 31 7 | 29 9 | 36 2 | 4 9 16 3 6 |

-----+-----------+------------------------------------+-----------+-----------+-----------+-----------+--------------------------+

m1 | 259 18 | 17 15 112 51 31 9 42 | 80 197 | 194 83 | 200 77 | 262 15 | 47 73 67 39 51 |

m2 | 41 2 | 2 1 24 8 2 2 4 | 7 36 | 35 8 | 32 11 | 41 2 | 4 12 19 2 6 |

-----+-----------+------------------------------------+-----------+-----------+-----------+-----------+--------------------------+

m1 | 281 20 | 18 16 130 55 31 10 41 | 82 219 | 218 83 | 219 82 | 284 17 | 46 76 82 41 56 |

m2 | 19 0 | 1 0 6 4 2 1 5 | 5 14 | 11 8 | 13 6 | 19 0 | 5 9 4 0 1 |

-----+-----------+------------------------------------+-----------+-----------+-----------+-----------+--------------------------+

m1 | 289 20 | 19 15 130 57 33 11 44 | 84 225 | 220 89 | 224 85 | 292 17 | 51 83 80 38 57 |

m2 | 11 0 | 0 1 6 2 0 0 2 | 3 8 | 9 2 | 8 3 | 11 0 | 0 2 6 3 0 |

-----+-----------+------------------------------------+-----------+-----------+-----------+-----------+--------------------------+

m1 | 274 18 | 19 15 129 54 28 11 36 | 76 216 | 216 76 | 210 82 | 275 17 | 41 80 81 38 52 |

m4 | 26 2 | 0 1 7 5 5 0 10 | 11 17 | 13 15 | 22 6 | 28 0 | 10 5 5 3 5 |

-----+-----------+------------------------------------+-----------+-----------+-----------+-----------+--------------------------+

m1 | 295 18 | 19 15 131 59 33 11 45 | 85 228 | 223 90 | 226 87 | 296 17 | 50 84 84 41 54 |

m2 | 5 2 | 0 1 5 0 0 0 1 | 2 5 | 6 1 | 6 1 | 7 0 | 1 1 2 0 3 |

-----+-----------+------------------------------------+-----------+-----------+-----------+-----------+--------------------------+

m1 | 293 20 | 19 16 136 58 33 11 40 | 81 232 | 228 85 | 226 87 | 296 17 | 48 85 82 41 57 |

m2 | 7 0 | 0 0 0 1 0 0 6 | 6 1 | 1 6 | 6 1 | 7 0 | 3 0 4 0 0 |

-----+-----------+------------------------------------+-----------+-----------+-----------+-----------+--------------------------+

m1 | 284 19 | 19 16 132 57 29 11 39 | 80 223 | 223 80 | 217 86 | 286 17 | 44 83 81 39 56 |

m2 | 16 1 | 0 0 4 2 4 0 7 | 7 10 | 6 11 | 15 2 | 17 0 | 7 2 5 2 1 |

-----+-----------+------------------------------------+-----------+-----------+-----------+-----------+--------------------------+

m1 | 293 19 | 19 16 131 58 32 11 45 | 86 226 | 223 89 | 225 87 | 295 17 | 50 83 83 41 55 |

m2 | 7 1 | 0 0 5 1 1 0 1 | 1 7 | 6 2 | 7 1 | 8 0 | 1 2 3 0 2 |

-----+-----------+------------------------------------+-----------+-----------+-----------+-----------+--------------------------+

m1 | 232 17 | 18 10 121 33 22 10 35 | 67 182 | 181 68 | 196 53 | 233 16 | 40 57 75 31 46 |

m7 | 42 0 | 0 3 8 16 10 0 5 | 10 32 | 27 15 | 21 21 | 42 0 | 7 20 7 5 3 |

m23 | 26 3 | 1 3 7 10 1 1 6 | 10 19 | 21 8 | 15 14 | 28 1 | 4 8 4 5 8 |

-----+-----------+------------------------------------+-----------+-----------+-----------+-----------+--------------------------+

m1 | 265 20 | 19 13 132 46 25 10 40 | 76 209 | 209 76 | 216 69 | 268 17 | 46 66 80 38 55 |

m2 | 35 0 | 0 3 4 13 8 1 6 | 11 24 | 20 15 | 16 19 | 35 0 | 5 19 6 3 2 |

-----+-----------+------------------------------------+-----------+-----------+-----------+-----------+--------------------------+

| m1 m2 | m2 m4 m5 m6 m8 m9 m11 | m1 m2 | m1 m2 | m1 m2 | m1 m2 | m2 m3 m4 m7 m8 |

| m1 m2 | m2 m4 m5 m6 m8 m9 m11 | m1 m2 | m1 m2 | m1 m2 | m1 m2 | m2 m3 m4 m7 m8 |

-----+-----------+------------------------------------+-----------+-----------+-----------+-----------+--------------------------+

m1 | 292 20 | 19 16 134 56 32 10 45 | 86 226 | 224 88 | 228 84 | 295 17 | 50 82 84 39 57 |

m2 | 8 0 | 0 0 2 3 1 1 1 | 1 7 | 5 3 | 4 4 | 8 0 | 1 3 2 2 0 |

-----+-----------+------------------------------------+-----------+-----------+-----------+-----------+--------------------------+

m1 | 263 17 | 18 13 126 43 31 9 40 | 77 203 | 199 81 | 213 67 | 264 16 | 46 74 76 34 50 |

m2 | 37 3 | 1 3 10 16 2 2 6 | 10 30 | 30 10 | 19 21 | 39 1 | 5 11 10 7 7 |

-----+-----------+------------------------------------+-----------+-----------+-----------+-----------+--------------------------+

m1 | 291 19 | 19 15 134 52 33 11 46 | 86 224 | 219 91 | 230 80 | 293 17 | 51 81 83 38 57 |

m2 | 9 1 | 0 1 2 7 0 0 0 | 1 9 | 10 0 | 2 8 | 10 0 | 0 4 3 3 0 |

-----+-----------+------------------------------------+-----------+-----------+-----------+-----------+--------------------------+

| m1 m2 | m2 m4 m5 m6 m8 m9 m11 | m1 m2 | m1 m2 | m1 m2 | m1 m2 | m2 m3 m4 m7 m8 |

| m4 m6 m7 m8 | m1 m2 | m1 m2 | m1 m2 | m1 m2 | m1 m2 m3 m6 | m1 m8 m11 m14 m26 m31 |

-----+---------------------+-----------+-----------+-----------+-----------+---------------------+-------------------------------+

m4 | 201 0 0 0 |

m6 | 0 23 0 0 |

m7 | 0 0 70 0 |

m8 | 0 0 0 26 |

-----+---------------------+-----------+

m1 | 201 22 3 8 | 234 0 |

m2 | 0 1 67 18 | 0 86 |

-----+---------------------+-----------+-----------+

m1 | 200 19 68 20 | 225 82 | 307 0 |

m2 | 1 4 2 6 | 9 4 | 0 13 |

-----+---------------------+-----------+-----------+-----------+

m1 | 4 23 69 9 | 36 69 | 94 11 | 105 0 |

m2 | 197 0 1 17 | 198 17 | 213 2 | 0 215 |

-----+---------------------+-----------+-----------+-----------+-----------+

m1 | 201 7 70 26 | 218 86 | 291 13 | 89 215 | 304 0 |

m2 | 0 16 0 0 | 16 0 | 16 0 | 16 0 | 0 16 |

-----+---------------------+-----------+-----------+-----------+-----------+---------------------+

m1 | 33 2 17 2 | 36 18 | 53 1 | 20 34 | 53 1 | 54 0 0 0 |

m2 | 10 1 1 0 | 11 1 | 12 0 | 2 10 | 11 1 | 0 12 0 0 |

m3 | 142 17 45 18 | 167 55 | 213 9 | 73 149 | 210 12 | 0 0 222 0 |

m6 | 16 3 7 6 | 20 12 | 29 3 | 10 22 | 30 2 | 0 0 0 32 |

-----+---------------------+-----------+-----------+-----------+-----------+---------------------+-------------------------------+

m1 | 47 5 14 6 | 55 17 | 69 3 | 23 49 | 69 3 | 12 0 55 5 | 72 0 0 0 0 0 |

m8 | 9 3 4 3 | 13 6 | 16 3 | 8 11 | 18 1 | 5 0 11 3 | 0 19 0 0 0 0 |

m11 | 38 5 16 4 | 45 18 | 60 3 | 24 39 | 60 3 | 6 5 46 6 | 0 0 63 0 0 0 |

m14 | 86 9 25 11 | 98 33 | 128 3 | 38 93 | 123 8 | 23 6 87 15 | 0 0 0 131 0 0 |

m26 | 15 0 8 2 | 16 9 | 24 1 | 8 17 | 25 0 | 5 0 17 3 | 0 0 0 0 25 0 |

m31 | 6 1 3 0 | 7 3 | 10 0 | 4 6 | 9 1 | 3 1 6 0 | 0 0 0 0 0 10 |

-----+---------------------+-----------+-----------+-----------+-----------+---------------------+-------------------------------+

m1 | 72 6 21 9 | 82 26 | 105 3 | 33 75 | 104 4 | 19 1 80 8 | 70 2 4 3 21 8 |

m2 | 129 17 49 17 | 152 60 | 202 10 | 72 140 | 200 12 | 35 11 142 24 | 2 17 59 128 4 2 |

-----+---------------------+-----------+-----------+-----------+-----------+---------------------+-------------------------------+

m1 | 62 8 16 7 | 73 20 | 89 4 | 28 65 | 88 5 | 14 1 70 8 | 68 10 4 3 1 7 |

m2 | 139 15 54 19 | 161 66 | 218 9 | 77 150 | 216 11 | 40 11 152 24 | 4 9 59 128 24 3 |

-----+---------------------+-----------+-----------+-----------+-----------+---------------------+-------------------------------+

m1 | 101 12 30 9 | 119 33 | 146 6 | 48 104 | 144 8 | 22 6 111 13 | 71 12 54 4 3 8 |

m2 | 100 11 40 17 | 115 53 | 161 7 | 57 111 | 160 8 | 32 6 111 19 | 1 7 9 127 22 2 |

-----+---------------------+-----------+-----------+-----------+-----------+---------------------+-------------------------------+

m1 | 201 22 53 22 | 230 68 | 290 8 | 85 213 | 282 16 | 51 12 206 29 | 69 15 55 128 22 9 |

m2 | 0 1 17 4 | 4 18 | 17 5 | 20 2 | 22 0 | 3 0 16 3 | 3 4 8 3 3 1 |

-----+---------------------+-----------+-----------+-----------+-----------+---------------------+-------------------------------+

m1 | 201 22 60 22 | 230 75 | 297 8 | 92 213 | 289 16 | 52 12 211 30 | 71 16 58 129 22 9 |

m2 | 0 1 10 4 | 4 11 | 10 5 | 13 2 | 15 0 | 2 0 11 2 | 1 3 5 2 3 1 |

-----+---------------------+-----------+-----------+-----------+-----------+---------------------+-------------------------------+

m1 | 155 18 59 20 | 180 72 | 242 10 | 85 167 | 239 13 | 43 12 170 27 | 4 19 63 131 25 10 |

m2 | 46 5 11 6 | 54 14 | 65 3 | 20 48 | 65 3 | 11 0 52 5 | 68 0 0 0 0 0 |

-----+---------------------+-----------+-----------+-----------+-----------+---------------------+-------------------------------+

m1 | 4 0 5 3 | 5 7 | 10 2 | 7 5 | 12 0 | 3 1 5 3 | 1 0 3 7 0 1 |

m2 | 139 7 29 16 | 152 39 | 184 7 | 41 150 | 188 3 | 29 7 133 22 | 47 9 37 80 14 4 |

m3 | 30 6 17 4 | 37 20 | 54 3 | 24 33 | 54 3 | 11 2 39 5 | 10 3 10 23 8 3 |

m4 | 20 8 19 3 | 30 20 | 49 1 | 31 19 | 42 8 | 9 1 39 1 | 13 4 13 15 3 2 |

m5 | 8 2 0 0 | 10 0 | 10 0 | 2 8 | 8 2 | 2 1 6 1 | 1 3 0 6 0 0 |

-----+---------------------+-----------+-----------+-----------+-----------+---------------------+-------------------------------+

m1 | 158 18 60 21 | 183 74 | 248 9 | 88 169 | 243 14 | 45 11 174 27 | 62 15 51 106 14 9 |

m5 | 22 3 7 5 | 28 9 | 34 3 | 11 26 | 37 0 | 6 0 26 5 | 6 4 8 12 7 0 |

m26 | 21 2 3 0 | 23 3 | 25 1 | 6 20 | 24 2 | 3 1 22 0 | 4 0 4 13 4 1 |

-----+---------------------+-----------+-----------+-----------+-----------+---------------------+-------------------------------+

m1 | 172 23 69 25 | 204 85 | 278 11 | 105 184 | 273 16 | 49 10 202 28 | 68 19 61 114 17 10 |

m2 | 29 0 1 1 | 30 1 | 29 2 | 0 31 | 31 0 | 5 2 20 4 | 4 0 2 17 8 0 |

-----+---------------------+-----------+-----------+-----------+-----------+---------------------+-------------------------------+

m1 | 190 22 67 26 | 222 83 | 292 13 | 101 204 | 289 16 | 54 12 209 30 | 69 19 62 125 20 10 |

m2 | 11 1 3 0 | 12 3 | 15 0 | 4 11 | 15 0 | 0 0 13 2 | 3 0 1 6 5 0 |

-----+---------------------+-----------+-----------+-----------+-----------+---------------------+-------------------------------+

| m4 m6 m7 m8 | m1 m2 | m1 m2 | m1 m2 | m1 m2 | m1 m2 m3 m6 | m1 m8 m11 m14 m26 m31 |

| m4 m6 m7 m8 | m1 m2 | m1 m2 | m1 m2 | m1 m2 | m1 m2 m3 m6 | m1 m8 m11 m14 m26 m31 |

-----+---------------------+-----------+-----------+-----------+-----------+---------------------+-------------------------------+

m1 | 194 22 67 23 | 226 80 | 294 12 | 101 205 | 290 16 | 53 12 212 29 | 71 17 57 127 24 10 |

m2 | 7 1 3 3 | 8 6 | 13 1 | 4 10 | 14 0 | 1 0 10 3 | 1 2 6 4 1 0 |

-----+---------------------+-----------+-----------+-----------+-----------+---------------------+-------------------------------+

m1 | 194 22 68 25 | 226 83 | 297 12 | 102 207 | 293 16 | 53 11 214 31 | 71 19 60 128 22 9 |

m2 | 7 1 2 1 | 8 3 | 10 1 | 3 8 | 11 0 | 1 1 8 1 | 1 0 3 3 3 1 |

-----+---------------------+-----------+-----------+-----------+-----------+---------------------+-------------------------------+

m1 | 116 8 31 8 | 129 34 | 161 2 | 45 118 | 158 5 | 31 9 114 9 | 42 11 31 63 9 7 |

m4 | 25 2 6 4 | 27 10 | 35 2 | 9 28 | 36 1 | 3 0 25 9 | 9 2 6 13 7 0 |

m5 | 16 2 4 2 | 19 5 | 23 1 | 8 16 | 22 2 | 4 0 16 4 | 5 2 4 10 2 1 |

m52 | 16 3 5 4 | 22 6 | 22 6 | 10 18 | 26 2 | 1 1 22 4 | 3 1 9 13 1 1 |

m54 | 3 4 11 2 | 7 13 | 18 2 | 15 5 | 17 3 | 6 0 11 3 | 2 1 4 12 0 1 |

m57 | 14 3 4 2 | 18 5 | 23 0 | 8 15 | 20 3 | 2 2 17 2 | 4 1 4 13 1 0 |

m60 | 11 1 9 4 | 12 13 | 25 0 | 10 15 | 25 0 | 7 0 17 1 | 7 1 5 7 5 0 |

-----+---------------------+-----------+-----------+-----------+-----------+---------------------+-------------------------------+

m1 | 199 21 65 25 | 230 80 | 298 12 | 97 213 | 296 14 | 52 11 218 29 | 69 19 61 127 25 9 |

m2 | 2 2 5 1 | 4 6 | 9 1 | 8 2 | 8 2 | 2 1 4 3 | 3 0 2 4 0 1 |

-----+---------------------+-----------+-----------+-----------+-----------+---------------------+-------------------------------+

m1 | 200 20 64 25 | 229 80 | 297 12 | 95 214 | 296 13 | 51 12 216 30 | 70 19 62 124 25 9 |

m2 | 1 3 6 1 | 5 6 | 10 1 | 10 1 | 8 3 | 3 0 6 2 | 2 0 1 7 0 1 |

-----+---------------------+-----------+-----------+-----------+-----------+---------------------+-------------------------------+

m1 | 197 21 52 21 | 228 63 | 280 11 | 85 206 | 275 16 | 48 12 201 30 | 67 19 53 121 21 10 |

m2 | 4 2 18 5 | 6 23 | 27 2 | 20 9 | 29 0 | 6 0 21 2 | 5 0 10 10 4 0 |

-----+---------------------+-----------+-----------+-----------+-----------+---------------------+-------------------------------+

m1 | 154 19 67 21 | 182 79 | 249 12 | 96 165 | 248 13 | 51 12 175 23 | 59 16 47 112 18 9 |

m2 | 47 4 3 5 | 52 7 | 58 1 | 9 50 | 56 3 | 3 0 47 9 | 13 3 16 19 7 1 |

-----+---------------------+-----------+-----------+-----------+-----------+---------------------+-------------------------------+

m1 | 185 21 69 23 | 215 83 | 285 13 | 101 197 | 284 14 | 52 12 205 29 | 70 17 55 121 25 10 |

m2 | 16 2 1 3 | 19 3 | 22 0 | 4 18 | 20 2 | 2 0 17 3 | 2 2 8 10 0 0 |

-----+---------------------+-----------+-----------+-----------+-----------+---------------------+-------------------------------+

m1 | 191 23 69 25 | 224 84 | 295 13 | 104 204 | 292 16 | 54 11 213 30 | 72 18 62 121 25 10 |

m2 | 10 0 1 1 | 10 2 | 12 0 | 1 11 | 12 0 | 0 1 9 2 | 0 1 1 10 0 0 |

-----+---------------------+-----------+-----------+-----------+-----------+---------------------+-------------------------------+

m1 | 193 18 61 18 | 216 74 | 287 3 | 86 204 | 277 13 | 51 12 200 27 | 67 18 55 117 23 10 |

m2 | 8 5 9 8 | 18 12 | 20 10 | 19 11 | 27 3 | 3 0 22 5 | 5 1 8 14 2 0 |

-----+---------------------+-----------+-----------+-----------+-----------+---------------------+-------------------------------+

m1 | 194 23 70 25 | 227 85 | 299 13 | 105 207 | 296 16 | 53 9 219 31 | 72 18 62 126 24 10 |

m2 | 7 0 0 1 | 7 1 | 8 0 | 0 8 | 8 0 | 1 3 3 1 | 0 1 1 5 1 0 |

-----+---------------------+-----------+-----------+-----------+-----------+---------------------+-------------------------------+

m1 | 192 22 69 26 | 224 85 | 296 13 | 103 206 | 293 16 | 52 12 213 32 | 70 19 63 125 24 8 |

m2 | 9 1 1 0 | 10 1 | 11 0 | 2 9 | 11 0 | 2 0 9 0 | 2 0 0 6 1 2 |

-----+---------------------+-----------+-----------+-----------+-----------+---------------------+-------------------------------+

m1 | 168 19 57 22 | 194 72 | 258 8 | 87 179 | 250 16 | 44 10 186 26 | 61 14 53 107 22 9 |

m4 | 14 0 2 0 | 14 2 | 16 0 | 2 14 | 16 0 | 2 0 12 2 | 2 1 4 9 0 0 |

m12 | 19 4 11 4 | 26 12 | 33 5 | 16 22 | 38 0 | 8 2 24 4 | 9 4 6 15 3 1 |

-----+---------------------+-----------+-----------+-----------+-----------+---------------------+-------------------------------+

m1 | 180 19 56 22 | 206 71 | 269 8 | 86 191 | 261 16 | 43 10 196 28 | 63 15 58 109 23 9 |

m2 | 21 4 14 4 | 28 15 | 38 5 | 19 24 | 43 0 | 11 2 26 4 | 9 4 5 22 2 1 |

-----+---------------------+-----------+-----------+-----------+-----------+---------------------+-------------------------------+

m1 | 187 22 67 25 | 218 83 | 290 11 | 102 199 | 285 16 | 53 12 207 29 | 69 19 58 121 24 10 |

m2 | 14 1 3 1 | 16 3 | 17 2 | 3 16 | 19 0 | 1 0 15 3 | 3 0 5 10 1 0 |

-----+---------------------+-----------+-----------+-----------+-----------+---------------------+-------------------------------+

m1 | 196 21 66 26 | 227 82 | 297 12 | 99 210 | 293 16 | 50 12 216 31 | 69 17 62 127 24 10 |

m2 | 5 2 4 0 | 7 4 | 10 1 | 6 5 | 11 0 | 4 0 6 1 | 3 2 1 4 1 0 |

-----+---------------------+-----------+-----------+-----------+-----------+---------------------+-------------------------------+

m1 | 181 21 66 24 | 212 80 | 281 11 | 98 194 | 277 15 | 46 10 208 28 | 69 17 58 119 21 8 |

m4 | 20 2 4 2 | 22 6 | 26 2 | 7 21 | 27 1 | 8 2 14 4 | 3 2 5 12 4 2 |

-----+---------------------+-----------+-----------+-----------+-----------+---------------------+-------------------------------+

m1 | 199 22 68 24 | 230 83 | 302 11 | 100 213 | 298 15 | 53 10 218 32 | 72 19 60 128 25 9 |

m2 | 2 1 2 2 | 4 3 | 5 2 | 5 2 | 6 1 | 1 2 4 0 | 0 0 3 3 0 1 |

-----+---------------------+-----------+-----------+-----------+-----------+---------------------+-------------------------------+

m1 | 196 23 69 25 | 228 85 | 300 13 | 103 210 | 297 16 | 54 11 217 31 | 72 19 60 128 24 10 |

m2 | 5 0 1 1 | 6 1 | 7 0 | 2 5 | 7 0 | 0 1 5 1 | 0 0 3 3 1 0 |

-----+---------------------+-----------+-----------+-----------+-----------+---------------------+-------------------------------+

m1 | 188 22 68 25 | 219 84 | 292 11 | 100 203 | 287 16 | 48 12 212 31 | 69 17 62 123 22 10 |

m2 | 13 1 2 1 | 15 2 | 15 2 | 5 12 | 17 0 | 6 0 10 1 | 3 2 1 8 3 0 |

-----+---------------------+-----------+-----------+-----------+-----------+---------------------+-------------------------------+

m1 | 199 22 67 24 | 230 82 | 301 11 | 100 212 | 297 15 | 53 12 217 30 | 71 19 60 129 24 9 |

m2 | 2 1 3 2 | 4 4 | 6 2 | 5 3 | 7 1 | 1 0 5 2 | 1 0 3 2 1 1 |

-----+---------------------+-----------+-----------+-----------+-----------+---------------------+-------------------------------+

m1 | 144 20 62 23 | 173 76 | 238 11 | 93 156 | 236 13 | 44 6 177 22 | 58 15 54 98 17 7 |

m7 | 38 1 3 0 | 40 2 | 41 1 | 5 37 | 41 1 | 8 2 29 3 | 4 4 6 23 5 0 |

m23 | 19 2 5 3 | 21 8 | 28 1 | 7 22 | 27 2 | 2 4 16 7 | 10 0 3 10 3 3 |

-----+---------------------+-----------+-----------+-----------+-----------+---------------------+-------------------------------+

m1 | 168 22 69 26 | 200 85 | 272 13 | 103 182 | 270 15 | 49 10 196 30 | 68 16 59 112 20 10 |

m2 | 33 1 1 0 | 34 1 | 35 0 | 2 33 | 34 1 | 5 2 26 2 | 4 3 4 19 5 0 |

-----+---------------------+-----------+-----------+-----------+-----------+---------------------+-------------------------------+

| m4 m6 m7 m8 | m1 m2 | m1 m2 | m1 m2 | m1 m2 | m1 m2 m3 m6 | m1 m8 m11 m14 m26 m31 |

| m4 m6 m7 m8 | m1 m2 | m1 m2 | m1 m2 | m1 m2 | m1 m2 m3 m6 | m1 m8 m11 m14 m26 m31 |

-----+---------------------+-----------+-----------+-----------+-----------+---------------------+-------------------------------+

m1 | 193 23 70 26 | 226 86 | 299 13 | 105 207 | 296 16 | 52 12 216 32 | 70 19 63 126 24 10 |

m2 | 8 0 0 0 | 8 0 | 8 0 | 0 8 | 8 0 | 2 0 6 0 | 2 0 0 5 1 0 |

-----+---------------------+-----------+-----------+-----------+-----------+---------------------+-------------------------------+

m1 | 170 21 66 23 | 201 79 | 267 13 | 99 181 | 266 14 | 49 9 198 24 | 60 18 60 112 22 8 |

m2 | 31 2 4 3 | 33 7 | 40 0 | 6 34 | 38 2 | 5 3 24 8 | 12 1 3 19 3 2 |

-----+---------------------+-----------+-----------+-----------+-----------+---------------------+-------------------------------+

m1 | 192 23 69 26 | 225 85 | 297 13 | 104 206 | 294 16 | 52 12 216 30 | 69 19 60 128 24 10 |

m2 | 9 0 1 0 | 9 1 | 10 0 | 1 9 | 10 0 | 2 0 6 2 | 3 0 3 3 1 0 |

-----+---------------------+-----------+-----------+-----------+-----------+---------------------+-------------------------------+

| m4 m6 m7 m8 | m1 m2 | m1 m2 | m1 m2 | m1 m2 | m1 m2 m3 m6 | m1 m8 m11 m14 m26 m31 |

| m1 m2 | m1 m2 | m1 m2 | m1 m2 | m1 m2 | m1 m2 | m1 m2 m3 m4 m5 | m1 m5 m26 |

-----+-----------+-----------+-----------+-----------+-----------+-----------+--------------------------+----------------+

m1 | 108 0 |

m2 | 0 212 |

-----+-----------+-----------+

m1 | 81 12 | 93 0 |

m2 | 27 200 | 0 227 |

-----+-----------+-----------+-----------+

m1 | 86 66 | 91 61 | 152 0 |

m2 | 22 146 | 2 166 | 0 168 |

-----+-----------+-----------+-----------+-----------+

m1 | 105 193 | 90 208 | 145 153 | 298 0 |

m2 | 3 19 | 3 19 | 7 15 | 0 22 |

-----+-----------+-----------+-----------+-----------+-----------+

m1 | 105 200 | 91 214 | 146 159 | 298 7 | 305 0 |

m2 | 3 12 | 2 13 | 6 9 | 0 15 | 0 15 |

-----+-----------+-----------+-----------+-----------+-----------+-----------+

m1 | 40 212 | 25 227 | 84 168 | 230 22 | 237 15 | 252 0 |

m2 | 68 0 | 68 0 | 68 0 | 68 0 | 68 0 | 0 68 |

-----+-----------+-----------+-----------+-----------+-----------+-----------+--------------------------+

m1 | 3 9 | 4 8 | 2 10 | 10 2 | 11 1 | 11 1 | 12 0 0 0 0 |

m2 | 66 125 | 58 133 | 96 95 | 187 4 | 187 4 | 144 47 | 0 191 0 0 0 |

m3 | 21 36 | 12 45 | 17 40 | 49 8 | 52 5 | 48 9 | 0 0 57 0 0 |

m4 | 17 33 | 15 35 | 33 17 | 42 8 | 45 5 | 40 10 | 0 0 0 50 0 |

m5 | 1 9 | 4 6 | 4 6 | 10 0 | 10 0 | 9 1 | 0 0 0 0 10 |

-----+-----------+-----------+-----------+-----------+-----------+-----------+--------------------------+----------------+

m1 | 87 170 | 80 177 | 129 128 | 240 17 | 246 11 | 199 58 | 11 147 43 46 10 | 257 0 0 |

m5 | 12 25 | 8 29 | 14 23 | 33 4 | 34 3 | 31 6 | 1 22 12 2 0 | 0 37 0 |

m26 | 9 17 | 5 21 | 9 17 | 25 1 | 25 1 | 22 4 | 0 22 2 2 0 | 0 0 26 |

-----+-----------+-----------+-----------+-----------+-----------+-----------+--------------------------+----------------+

m1 | 97 192 | 89 200 | 145 144 | 268 21 | 275 14 | 225 64 | 11 167 51 50 10 | 251 27 11 |

m2 | 11 20 | 4 27 | 7 24 | 30 1 | 30 1 | 27 4 | 1 24 6 0 0 | 6 10 15 |

-----+-----------+-----------+-----------+-----------+-----------+-----------+--------------------------+----------------+

m1 | 101 204 | 90 215 | 148 157 | 284 21 | 291 14 | 240 65 | 12 180 53 50 10 | 250 32 23 |

m2 | 7 8 | 3 12 | 4 11 | 14 1 | 14 1 | 12 3 | 0 11 4 0 0 | 7 5 3 |

-----+-----------+-----------+-----------+-----------+-----------+-----------+--------------------------+----------------+

m1 | 107 199 | 92 214 | 145 161 | 286 20 | 292 14 | 239 67 | 11 180 57 48 10 | 256 24 26 |

m2 | 1 13 | 1 13 | 7 7 | 12 2 | 13 1 | 13 1 | 1 11 0 2 0 | 1 13 0 |

-----+-----------+-----------+-----------+-----------+-----------+-----------+--------------------------+----------------+

m1 | 102 207 | 90 219 | 147 162 | 288 21 | 295 14 | 242 67 | 11 186 53 49 10 | 255 33 21 |

m2 | 6 5 | 3 8 | 5 6 | 10 1 | 10 1 | 10 1 | 1 5 4 1 0 | 2 4 5 |

-----+-----------+-----------+-----------+-----------+-----------+-----------+--------------------------+----------------+

m1 | 61 102 | 57 106 | 91 72 | 153 10 | 155 8 | 123 40 | 3 103 16 33 8 | 128 19 16 |

m4 | 17 20 | 9 28 | 14 23 | 36 1 | 37 0 | 29 8 | 2 25 9 1 0 | 29 6 2 |

m5 | 8 16 | 7 17 | 11 13 | 23 1 | 24 0 | 19 5 | 0 12 9 3 0 | 23 0 1 |

m52 | 5 23 | 5 23 | 12 16 | 24 4 | 25 3 | 25 3 | 2 12 9 5 0 | 24 2 2 |

m54 | 1 19 | 3 17 | 4 16 | 15 5 | 16 4 | 19 1 | 4 2 11 2 1 | 17 3 0 |

m57 | 5 18 | 4 19 | 7 16 | 22 1 | 23 0 | 19 4 | 0 16 2 4 1 | 16 3 4 |

m60 | 11 14 | 8 17 | 13 12 | 25 0 | 25 0 | 18 7 | 1 21 1 2 0 | 20 4 1 |

-----+-----------+-----------+-----------+-----------+-----------+-----------+--------------------------+----------------+

m1 | 106 204 | 90 220 | 150 160 | 292 18 | 297 13 | 244 66 | 7 191 53 50 9 | 247 37 26 |

m2 | 2 8 | 3 7 | 2 8 | 6 4 | 8 2 | 8 2 | 5 0 4 0 1 | 10 0 0 |

-----+-----------+-----------+-----------+-----------+-----------+-----------+--------------------------+----------------+

m1 | 105 204 | 90 219 | 150 159 | 288 21 | 294 15 | 242 67 | 9 188 54 48 10 | 247 37 25 |

m2 | 3 8 | 3 8 | 2 9 | 10 1 | 11 0 | 10 1 | 3 3 3 2 0 | 10 0 1 |

-----+-----------+-----------+-----------+-----------+-----------+-----------+--------------------------+----------------+

m1 | 99 192 | 87 204 | 141 150 | 274 17 | 279 12 | 228 63 | 12 175 45 49 10 | 232 33 26 |

m2 | 9 20 | 6 23 | 11 18 | 24 5 | 26 3 | 24 5 | 0 16 12 1 0 | 25 4 0 |

-----+-----------+-----------+-----------+-----------+-----------+-----------+--------------------------+----------------+

m1 | 87 174 | 79 182 | 123 138 | 240 21 | 246 15 | 205 56 | 12 152 42 45 10 | 208 31 22 |

m2 | 21 38 | 14 45 | 29 30 | 58 1 | 59 0 | 47 12 | 0 39 15 5 0 | 49 6 4 |

-----+-----------+-----------+-----------+-----------+-----------+-----------+--------------------------+----------------+

m1 | 106 192 | 89 209 | 141 157 | 276 22 | 283 15 | 232 66 | 12 178 52 46 10 | 236 37 25 |

m2 | 2 20 | 4 18 | 11 11 | 22 0 | 22 0 | 20 2 | 0 13 5 4 0 | 21 0 1 |

-----+-----------+-----------+-----------+-----------+-----------+-----------+--------------------------+----------------+

| m1 m2 | m1 m2 | m1 m2 | m1 m2 | m1 m2 | m1 m2 | m1 m2 m3 m4 m5 | m1 m5 m26 |

| m1 m2 | m1 m2 | m1 m2 | m1 m2 | m1 m2 | m1 m2 | m1 m2 m3 m4 m5 | m1 m5 m26 |

-----+-----------+-----------+-----------+-----------+-----------+-----------+--------------------------+----------------+

m1 | 108 200 | 93 215 | 151 157 | 287 21 | 293 15 | 240 68 | 12 182 55 49 10 | 250 34 24 |

m2 | 0 12 | 0 12 | 1 11 | 11 1 | 12 0 | 12 0 | 0 9 2 1 0 | 7 3 2 |

-----+-----------+-----------+-----------+-----------+-----------+-----------+--------------------------+----------------+

m1 | 102 188 | 87 203 | 141 149 | 275 15 | 281 9 | 227 63 | 9 182 44 45 10 | 235 32 23 |

m2 | 6 24 | 6 24 | 11 19 | 23 7 | 24 6 | 25 5 | 3 9 13 5 0 | 22 5 3 |

-----+-----------+-----------+-----------+-----------+-----------+-----------+--------------------------+----------------+

m1 | 107 205 | 92 220 | 150 162 | 290 22 | 297 15 | 244 68 | 12 185 55 50 10 | 251 36 25 |

m2 | 1 7 | 1 7 | 2 6 | 8 0 | 8 0 | 8 0 | 0 6 2 0 0 | 6 1 1 |

-----+-----------+-----------+-----------+-----------+-----------+-----------+--------------------------+----------------+

m1 | 105 204 | 91 218 | 149 160 | 287 22 | 294 15 | 243 66 | 12 184 53 50 10 | 248 35 26 |

m2 | 3 8 | 2 9 | 3 8 | 11 0 | 11 0 | 9 2 | 0 7 4 0 0 | 9 2 0 |

-----+-----------+-----------+-----------+-----------+-----------+-----------+--------------------------+----------------+

m1 | 94 172 | 81 185 | 128 138 | 248 18 | 255 11 | 209 57 | 9 154 48 47 8 | 219 28 19 |

m4 | 3 13 | 2 14 | 6 10 | 16 0 | 16 0 | 14 2 | 0 11 2 1 2 | 13 3 0 |

m12 | 11 27 | 10 28 | 18 20 | 34 4 | 34 4 | 29 9 | 3 26 7 2 0 | 25 6 7 |

-----+-----------+-----------+-----------+-----------+-----------+-----------+--------------------------+----------------+

m1 | 98 179 | 83 194 | 135 142 | 259 18 | 266 11 | 218 59 | 9 160 50 48 10 | 228 30 19 |

m2 | 10 33 | 10 33 | 17 26 | 39 4 | 39 4 | 34 9 | 3 31 7 2 0 | 29 7 7 |

-----+-----------+-----------+-----------+-----------+-----------+-----------+--------------------------+----------------+

m1 | 105 196 | 90 211 | 143 158 | 280 21 | 287 14 | 236 65 | 12 175 56 49 9 | 242 33 26 |

m2 | 3 16 | 3 16 | 9 10 | 18 1 | 18 1 | 16 3 | 0 16 1 1 1 | 15 4 0 |

-----+-----------+-----------+-----------+-----------+-----------+-----------+--------------------------+----------------+

m1 | 103 206 | 90 219 | 148 161 | 287 22 | 294 15 | 244 65 | 12 184 54 50 9 | 248 37 24 |

m2 | 5 6 | 3 8 | 4 7 | 11 0 | 11 0 | 8 3 | 0 7 3 0 1 | 9 0 2 |

-----+-----------+-----------+-----------+-----------+-----------+-----------+--------------------------+----------------+

m1 | 97 195 | 89 203 | 146 146 | 273 19 | 279 13 | 227 65 | 10 176 47 50 9 | 240 32 20 |

m4 | 11 17 | 4 24 | 6 22 | 25 3 | 26 2 | 25 3 | 2 15 10 0 1 | 17 5 6 |

-----+-----------+-----------+-----------+-----------+-----------+-----------+--------------------------+----------------+

m1 | 107 206 | 92 221 | 151 162 | 293 20 | 300 13 | 245 68 | 9 190 55 50 9 | 252 37 24 |

m2 | 1 6 | 1 6 | 1 6 | 5 2 | 5 2 | 7 0 | 3 1 2 0 1 | 5 0 2 |

-----+-----------+-----------+-----------+-----------+-----------+-----------+--------------------------+----------------+

m1 | 106 207 | 92 221 | 150 163 | 291 22 | 298 15 | 245 68 | 10 187 56 50 10 | 253 34 26 |

m2 | 2 5 | 1 6 | 2 5 | 7 0 | 7 0 | 7 0 | 2 4 1 0 0 | 4 3 0 |

-----+-----------+-----------+-----------+-----------+-----------+-----------+--------------------------+----------------+

m1 | 101 202 | 90 213 | 148 155 | 281 22 | 288 15 | 238 65 | 12 180 51 50 10 | 247 34 22 |

m2 | 7 10 | 3 14 | 4 13 | 17 0 | 17 0 | 14 3 | 0 11 6 0 0 | 10 3 4 |

-----+-----------+-----------+-----------+-----------+-----------+-----------+--------------------------+----------------+

m1 | 104 208 | 91 221 | 150 162 | 292 20 | 298 14 | 245 67 | 10 190 52 50 10 | 251 35 26 |

m2 | 4 4 | 2 6 | 2 6 | 6 2 | 7 1 | 7 1 | 2 1 5 0 0 | 6 2 0 |

-----+-----------+-----------+-----------+-----------+-----------+-----------+--------------------------+----------------+

m1 | 82 167 | 74 175 | 127 122 | 229 20 | 236 13 | 195 54 | 10 146 39 45 9 | 204 25 20 |

m7 | 10 32 | 6 36 | 12 30 | 42 0 | 42 0 | 38 4 | 1 26 11 3 1 | 29 9 4 |

m23 | 16 13 | 13 16 | 13 16 | 27 2 | 27 2 | 19 10 | 1 19 7 2 0 | 24 3 2 |

-----+-----------+-----------+-----------+-----------+-----------+-----------+--------------------------+----------------+

m1 | 98 187 | 87 198 | 142 143 | 263 22 | 270 15 | 221 64 | 12 171 45 48 9 | 233 29 23 |

m2 | 10 25 | 6 29 | 10 25 | 35 0 | 35 0 | 31 4 | 0 20 12 2 1 | 24 8 3 |

-----+-----------+-----------+-----------+-----------+-----------+-----------+--------------------------+----------------+

m1 | 105 207 | 91 221 | 150 162 | 290 22 | 297 15 | 246 66 | 12 185 55 50 10 | 250 36 26 |

m2 | 3 5 | 2 6 | 2 6 | 8 0 | 8 0 | 6 2 | 0 6 2 0 0 | 7 1 0 |

-----+-----------+-----------+-----------+-----------+-----------+-----------+--------------------------+----------------+

m1 | 90 190 | 76 204 | 136 144 | 259 21 | 265 15 | 224 56 | 10 162 50 48 10 | 224 34 22 |

m2 | 18 22 | 17 23 | 16 24 | 39 1 | 40 0 | 28 12 | 2 29 7 2 0 | 33 3 4 |

-----+-----------+-----------+-----------+-----------+-----------+-----------+--------------------------+----------------+

m1 | 104 206 | 90 220 | 146 164 | 288 22 | 295 15 | 245 65 | 12 184 54 50 10 | 251 34 25 |

m2 | 4 6 | 3 7 | 6 4 | 10 0 | 10 0 | 7 3 | 0 7 3 0 0 | 6 3 1 |

-----+-----------+-----------+-----------+-----------+-----------+-----------+--------------------------+----------------+

| m1 m2 | m1 m2 | m1 m2 | m1 m2 | m1 m2 | m1 m2 | m1 m2 m3 m4 m5 | m1 m5 m26 |

| m1 m2 | m1 m2 | m1 m2 | m1 m2 | m1 m4 m5 m52 m54 m57 m60 | m1 m2 | m1 m2 | m1 m2 |

-----+-----------+-----------+-----------+-----------+------------------------------------+-----------+-----------+-----------+

m1 | 289 0 |

m2 | 0 31 |

-----+-----------+-----------+

m1 | 283 22 | 305 0 |

m2 | 6 9 | 0 15 |

-----+-----------+-----------+-----------+

m1 | 276 30 | 292 14 | 306 0 |

m2 | 13 1 | 13 1 | 0 14 |

-----+-----------+-----------+-----------+-----------+

m1 | 280 29 | 294 15 | 295 14 | 309 0 |

m2 | 9 2 | 11 0 | 11 0 | 0 11 |

-----+-----------+-----------+-----------+-----------+------------------------------------+

| m1 m2 | m1 m2 | m1 m2 | m1 m2 | m1 m4 m5 m52 m54 m57 m60 | m1 m2 | m1 m2 | m1 m2 |

| m1 m2 | m1 m2 | m1 m2 | m1 m2 | m1 m4 m5 m52 m54 m57 m60 | m1 m2 | m1 m2 | m1 m2 |

-----+-----------+-----------+-----------+-----------+------------------------------------+-----------+-----------+-----------+

m1 | 146 17 | 154 9 | 156 7 | 157 6 | 163 0 0 0 0 0 0 |

m4 | 32 5 | 34 3 | 36 1 | 36 1 | 0 37 0 0 0 0 0 |

m5 | 23 1 | 24 0 | 24 0 | 24 0 | 0 0 24 0 0 0 0 |

m52 | 26 2 | 28 0 | 26 2 | 27 1 | 0 0 0 28 0 0 0 |

m54 | 19 1 | 20 0 | 20 0 | 18 2 | 0 0 0 0 20 0 0 |

m57 | 19 4 | 21 2 | 22 1 | 23 0 | 0 0 0 0 0 23 0 |

m60 | 24 1 | 24 1 | 22 3 | 24 1 | 0 0 0 0 0 0 25 |

-----+-----------+-----------+-----------+-----------+------------------------------------+-----------+

m1 | 279 31 | 295 15 | 296 14 | 299 11 | 163 35 24 27 14 22 25 | 310 0 |

m2 | 10 0 | 10 0 | 10 0 | 10 0 | 0 2 0 1 6 1 0 | 0 10 |

-----+-----------+-----------+-----------+-----------+------------------------------------+-----------+-----------+

m1 | 278 31 | 294 15 | 295 14 | 298 11 | 163 34 23 25 18 21 25 | 300 9 | 309 0 |

m2 | 11 0 | 11 0 | 11 0 | 11 0 | 0 3 1 3 2 2 0 | 10 1 | 0 11 |

-----+-----------+-----------+-----------+-----------+------------------------------------+-----------+-----------+-----------+

m1 | 260 31 | 277 14 | 278 13 | 282 9 | 163 37 23 25 12 21 10 | 281 10 | 280 11 | 291 0 |

m2 | 29 0 | 28 1 | 28 1 | 27 2 | 0 0 1 3 8 2 15 | 29 0 | 29 0 | 0 29 |

-----+-----------+-----------+-----------+-----------+------------------------------------+-----------+-----------+-----------+

m1 | 236 25 | 250 11 | 248 13 | 252 9 | 159 10 7 23 19 20 23 | 251 10 | 251 10 | 234 27 |

m2 | 53 6 | 55 4 | 58 1 | 57 2 | 4 27 17 5 1 3 2 | 59 0 | 58 1 | 57 2 |

-----+-----------+-----------+-----------+-----------+------------------------------------+-----------+-----------+-----------+

m1 | 267 31 | 283 15 | 284 14 | 288 10 | 161 37 12 24 19 20 25 | 288 10 | 287 11 | 269 29 |

m2 | 22 0 | 22 0 | 22 0 | 21 1 | 2 0 12 4 1 3 0 | 22 0 | 22 0 | 22 0 |

-----+-----------+-----------+-----------+-----------+------------------------------------+-----------+-----------+-----------+

m1 | 280 28 | 294 14 | 295 13 | 297 11 | 162 36 23 26 20 16 25 | 298 10 | 297 11 | 279 29 |

m2 | 9 3 | 11 1 | 11 1 | 12 0 | 1 1 1 2 0 7 0 | 12 0 | 12 0 | 12 0 |

-----+-----------+-----------+-----------+-----------+------------------------------------+-----------+-----------+-----------+

m1 | 262 28 | 276 14 | 278 12 | 280 10 | 163 34 22 14 11 21 25 | 282 8 | 282 8 | 270 20 |

m2 | 27 3 | 29 1 | 28 2 | 29 1 | 0 3 2 14 9 2 0 | 28 2 | 27 3 | 21 9 |

-----+-----------+-----------+-----------+-----------+------------------------------------+-----------+-----------+-----------+

m1 | 282 30 | 297 15 | 298 14 | 301 11 | 162 35 23 27 20 21 24 | 302 10 | 301 11 | 283 29 |

m2 | 7 1 | 8 0 | 8 0 | 8 0 | 1 2 1 1 0 2 1 | 8 0 | 8 0 | 8 0 |

-----+-----------+-----------+-----------+-----------+------------------------------------+-----------+-----------+-----------+

m1 | 278 31 | 296 13 | 296 13 | 298 11 | 160 37 23 26 20 22 21 | 299 10 | 298 11 | 281 28 |

m2 | 11 0 | 9 2 | 10 1 | 11 0 | 3 0 1 2 0 1 4 | 11 0 | 11 0 | 10 1 |

-----+-----------+-----------+-----------+-----------+------------------------------------+-----------+-----------+-----------+

m1 | 245 21 | 256 10 | 259 7 | 256 10 | 130 31 23 22 18 22 20 | 258 8 | 257 9 | 240 26 |

m4 | 15 1 | 14 2 | 12 4 | 16 0 | 11 1 0 2 0 1 1 | 16 0 | 16 0 | 15 1 |

m12 | 29 9 | 35 3 | 35 3 | 37 1 | 22 5 1 4 2 0 4 | 36 2 | 36 2 | 36 2 |

-----+-----------+-----------+-----------+-----------+------------------------------------+-----------+-----------+-----------+

m1 | 256 21 | 266 11 | 267 10 | 267 10 | 138 33 22 24 17 22 21 | 269 8 | 268 9 | 251 26 |

m2 | 33 10 | 39 4 | 39 4 | 42 1 | 25 4 2 4 3 1 4 | 41 2 | 41 2 | 40 3 |

-----+-----------+-----------+-----------+-----------+------------------------------------+-----------+-----------+-----------+

m1 | 273 28 | 289 12 | 292 9 | 290 11 | 150 37 24 24 20 22 24 | 291 10 | 290 11 | 273 28 |

m2 | 16 3 | 16 3 | 14 5 | 19 0 | 13 0 0 4 0 1 1 | 19 0 | 19 0 | 18 1 |

-----+-----------+-----------+-----------+-----------+------------------------------------+-----------+-----------+-----------+

m1 | 280 29 | 294 15 | 295 14 | 298 11 | 158 35 22 28 19 22 25 | 299 10 | 298 11 | 281 28 |

m2 | 9 2 | 11 0 | 11 0 | 11 0 | 5 2 2 0 1 1 0 | 11 0 | 11 0 | 10 1 |

-----+-----------+-----------+-----------+-----------+------------------------------------+-----------+-----------+-----------+

m1 | 269 23 | 280 12 | 278 14 | 285 7 | 152 33 22 24 19 20 22 | 285 7 | 282 10 | 265 27 |

m4 | 20 8 | 25 3 | 28 0 | 24 4 | 11 4 2 4 1 3 3 | 25 3 | 27 1 | 26 2 |

-----+-----------+-----------+-----------+-----------+------------------------------------+-----------+-----------+-----------+

m1 | 283 30 | 298 15 | 299 14 | 302 11 | 163 36 24 25 19 21 25 | 306 7 | 304 9 | 284 29 |

m2 | 6 1 | 7 0 | 7 0 | 7 0 | 0 1 0 3 1 2 0 | 4 3 | 5 2 | 7 0 |

-----+-----------+-----------+-----------+-----------+------------------------------------+-----------+-----------+-----------+

m1 | 284 29 | 299 14 | 299 14 | 304 9 | 159 36 24 27 20 23 24 | 303 10 | 303 10 | 285 28 |

m2 | 5 2 | 6 1 | 7 0 | 5 2 | 4 1 0 1 0 0 1 | 7 0 | 6 1 | 6 1 |

-----+-----------+-----------+-----------+-----------+------------------------------------+-----------+-----------+-----------+

m1 | 278 25 | 290 13 | 289 14 | 293 10 | 154 34 23 26 20 23 23 | 293 10 | 293 10 | 274 29 |

m2 | 11 6 | 15 2 | 17 0 | 16 1 | 9 3 1 2 0 0 2 | 17 0 | 16 1 | 17 0 |

-----+-----------+-----------+-----------+-----------+------------------------------------+-----------+-----------+-----------+

m1 | 281 31 | 297 15 | 298 14 | 302 10 | 163 37 23 24 18 23 24 | 303 9 | 302 10 | 286 26 |

m2 | 8 0 | 8 0 | 8 0 | 7 1 | 0 0 1 4 2 0 1 | 7 1 | 7 1 | 5 3 |

-----+-----------+-----------+-----------+-----------+------------------------------------+-----------+-----------+-----------+

m1 | 228 21 | 240 9 | 236 13 | 241 8 | 120 29 19 25 16 20 20 | 241 8 | 240 9 | 223 26 |

m7 | 36 6 | 39 3 | 41 1 | 40 2 | 25 4 2 2 2 3 4 | 41 1 | 42 0 | 41 1 |

m23 | 25 4 | 26 3 | 29 0 | 28 1 | 18 4 3 1 2 0 1 | 28 1 | 27 2 | 27 2 |

-----+-----------+-----------+-----------+-----------+------------------------------------+-----------+-----------+-----------+

m1 | 260 25 | 273 12 | 272 13 | 276 9 | 142 31 23 27 19 20 23 | 275 10 | 274 11 | 256 29 |

m2 | 29 6 | 32 3 | 34 1 | 33 2 | 21 6 1 1 1 3 2 | 35 0 | 35 0 | 35 0 |

-----+-----------+-----------+-----------+-----------+------------------------------------+-----------+-----------+-----------+

m1 | 281 31 | 297 15 | 298 14 | 301 11 | 156 36 24 28 20 23 25 | 302 10 | 301 11 | 283 29 |

m2 | 8 0 | 8 0 | 8 0 | 8 0 | 7 1 0 0 0 0 0 | 8 0 | 8 0 | 8 0 |

-----+-----------+-----------+-----------+-----------+------------------------------------+-----------+-----------+-----------+

m1 | 254 26 | 267 13 | 266 14 | 270 10 | 134 31 21 27 20 23 24 | 271 9 | 271 9 | 252 28 |

m2 | 35 5 | 38 2 | 40 0 | 39 1 | 29 6 3 1 0 0 1 | 39 1 | 38 2 | 39 1 |

-----+-----------+-----------+-----------+-----------+------------------------------------+-----------+-----------+-----------+

| m1 m2 | m1 m2 | m1 m2 | m1 m2 | m1 m4 m5 m52 m54 m57 m60 | m1 m2 | m1 m2 | m1 m2 |

| m1 m2 | m1 m2 | m1 m2 | m1 m2 | m1 m4 m5 m52 m54 m57 m60 | m1 m2 | m1 m2 | m1 m2 |

-----+-----------+-----------+-----------+-----------+------------------------------------+-----------+-----------+-----------+

m1 | 280 30 | 296 14 | 297 13 | 299 11 | 156 36 23 28 20 22 25 | 300 10 | 299 11 | 281 29 |

m2 | 9 1 | 9 1 | 9 1 | 10 0 | 7 1 1 0 0 1 0 | 10 0 | 10 0 | 10 0 |

-----+-----------+-----------+-----------+-----------+------------------------------------+-----------+-----------+-----------+

| m1 m2 | m1 m2 | m1 m2 | m1 m2 | m1 m4 m5 m52 m54 m57 m60 | m1 m2 | m1 m2 | m1 m2 |

| m1 m2 | m1 m2 | m1 m2 | m1 m2 | m1 m2 | m1 m2 | m1 m4 m12 | m1 m2 | m1 m2 | m1 m2 |

-----+-----------+-----------+-----------+-----------+-----------+-----------+----------------+-----------+-----------+-----------+

m1 | 261 0 |

m2 | 0 59 |

-----+-----------+-----------+

m1 | 259 39 | 298 0 |

m2 | 2 20 | 0 22 |

-----+-----------+-----------+-----------+

m1 | 250 58 | 286 22 | 308 0 |

m2 | 11 1 | 12 0 | 0 12 |

-----+-----------+-----------+-----------+-----------+

m1 | 234 56 | 269 21 | 278 12 | 290 0 |

m2 | 27 3 | 29 1 | 30 0 | 0 30 |

-----+-----------+-----------+-----------+-----------+-----------+

m1 | 253 59 | 290 22 | 303 9 | 282 30 | 312 0 |

m2 | 8 0 | 8 0 | 5 3 | 8 0 | 0 8 |

-----+-----------+-----------+-----------+-----------+-----------+-----------+

m1 | 255 54 | 290 19 | 297 12 | 279 30 | 301 8 | 309 0 |

m2 | 6 5 | 8 3 | 11 0 | 11 0 | 11 0 | 0 11 |

-----+-----------+-----------+-----------+-----------+-----------+-----------+----------------+

m1 | 212 54 | 244 22 | 256 10 | 244 22 | 258 8 | 257 9 | 266 0 0 |

m4 | 15 1 | 16 0 | 15 1 | 14 2 | 16 0 | 16 0 | 0 16 0 |

m12 | 34 4 | 38 0 | 37 1 | 32 6 | 38 0 | 36 2 | 0 0 38 |

-----+-----------+-----------+-----------+-----------+-----------+-----------+----------------+-----------+

m1 | 221 56 | 255 22 | 267 10 | 254 23 | 269 8 | 268 9 | 261 14 2 | 277 0 |

m2 | 40 3 | 43 0 | 41 2 | 36 7 | 43 0 | 41 2 | 5 2 36 | 0 43 |

-----+-----------+-----------+-----------+-----------+-----------+-----------+----------------+-----------+-----------+

m1 | 242 59 | 279 22 | 289 12 | 275 26 | 293 8 | 290 11 | 264 3 34 | 265 36 | 301 0 |

m2 | 19 0 | 19 0 | 19 0 | 15 4 | 19 0 | 19 0 | 2 13 4 | 12 7 | 0 19 |

-----+-----------+-----------+-----------+-----------+-----------+-----------+----------------+-----------+-----------+-----------+

m1 | 252 57 | 287 22 | 298 11 | 280 29 | 301 8 | 298 11 | 262 14 33 | 273 36 | 291 18 | 309 0 |

m2 | 9 2 | 11 0 | 10 1 | 10 1 | 11 0 | 11 0 | 4 2 5 | 4 7 | 10 1 | 0 11 |

-----+-----------+-----------+-----------+-----------+-----------+-----------+----------------+-----------+-----------+-----------+

m1 | 237 55 | 270 22 | 281 11 | 266 26 | 285 7 | 282 10 | 245 14 33 | 254 38 | 273 19 | 283 9 |

m4 | 24 4 | 28 0 | 27 1 | 24 4 | 27 1 | 27 1 | 21 2 5 | 23 5 | 28 0 | 26 2 |

-----+-----------+-----------+-----------+-----------+-----------+-----------+----------------+-----------+-----------+-----------+

m1 | 254 59 | 291 22 | 301 12 | 286 27 | 306 7 | 302 11 | 262 16 35 | 273 40 | 294 19 | 302 11 |

m2 | 7 0 | 7 0 | 7 0 | 4 3 | 6 1 | 7 0 | 4 0 3 | 4 3 | 7 0 | 7 0 |

-----+-----------+-----------+-----------+-----------+-----------+-----------+----------------+-----------+-----------+-----------+

m1 | 255 58 | 291 22 | 301 12 | 283 30 | 305 8 | 302 11 | 260 16 37 | 271 42 | 294 19 | 302 11 |

m2 | 6 1 | 7 0 | 7 0 | 7 0 | 7 0 | 7 0 | 6 0 1 | 6 1 | 7 0 | 7 0 |

-----+-----------+-----------+-----------+-----------+-----------+-----------+----------------+-----------+-----------+-----------+

m1 | 246 57 | 281 22 | 291 12 | 276 27 | 295 8 | 292 11 | 254 15 34 | 264 39 | 284 19 | 294 9 |

m2 | 15 2 | 17 0 | 17 0 | 14 3 | 17 0 | 17 0 | 12 1 4 | 13 4 | 17 0 | 15 2 |

-----+-----------+-----------+-----------+-----------+-----------+-----------+----------------+-----------+-----------+-----------+

m1 | 253 59 | 290 22 | 300 12 | 286 26 | 304 8 | 302 10 | 258 16 38 | 269 43 | 293 19 | 301 11 |

m2 | 8 0 | 8 0 | 8 0 | 4 4 | 8 0 | 7 1 | 8 0 0 | 8 0 | 8 0 | 8 0 |

-----+-----------+-----------+-----------+-----------+-----------+-----------+----------------+-----------+-----------+-----------+

m1 | 199 50 | 227 22 | 241 8 | 224 25 | 245 4 | 239 10 | 207 10 32 | 213 36 | 234 15 | 240 9 |

m7 | 38 4 | 42 0 | 38 4 | 38 4 | 39 3 | 41 1 | 34 5 3 | 39 3 | 39 3 | 41 1 |

m23 | 24 5 | 29 0 | 29 0 | 28 1 | 28 1 | 29 0 | 25 1 3 | 25 4 | 28 1 | 28 1 |

-----+-----------+-----------+-----------+-----------+-----------+-----------+----------------+-----------+-----------+-----------+

m1 | 231 54 | 263 22 | 276 9 | 257 28 | 281 4 | 274 11 | 238 11 36 | 244 41 | 269 16 | 275 10 |

m2 | 30 5 | 35 0 | 32 3 | 33 2 | 31 4 | 35 0 | 28 5 2 | 33 2 | 32 3 | 34 1 |

-----+-----------+-----------+-----------+-----------+-----------+-----------+----------------+-----------+-----------+-----------+

m1 | 253 59 | 290 22 | 300 12 | 282 30 | 305 7 | 301 11 | 261 14 37 | 271 41 | 295 17 | 301 11 |

m2 | 8 0 | 8 0 | 8 0 | 8 0 | 7 1 | 8 0 | 5 2 1 | 6 2 | 6 2 | 8 0 |

-----+-----------+-----------+-----------+-----------+-----------+-----------+----------------+-----------+-----------+-----------+

m1 | 226 54 | 258 22 | 269 11 | 250 30 | 274 6 | 269 11 | 232 14 34 | 243 37 | 263 17 | 270 10 |

m2 | 35 5 | 40 0 | 39 1 | 40 0 | 38 2 | 40 0 | 34 2 4 | 34 6 | 38 2 | 39 1 |

-----+-----------+-----------+-----------+-----------+-----------+-----------+----------------+-----------+-----------+-----------+

m1 | 251 59 | 288 22 | 299 11 | 280 30 | 303 7 | 299 11 | 257 16 37 | 267 43 | 291 19 | 300 10 |

m2 | 10 0 | 10 0 | 9 1 | 10 0 | 9 1 | 10 0 | 9 0 1 | 10 0 | 10 0 | 9 1 |

-----+-----------+-----------+-----------+-----------+-----------+-----------+----------------+-----------+-----------+-----------+

| m1 m2 | m1 m2 | m1 m2 | m1 m2 | m1 m2 | m1 m2 | m1 m4 m12 | m1 m2 | m1 m2 | m1 m2 |

| m1 m4 | m1 m2 | m1 m2 | m1 m2 | m1 m2 | m1 m7 m23 | m1 m2 | m1 m2 | m1 m2 | m1 m2 |

-----+-----------+-----------+-----------+-----------+-----------+----------------+-----------+-----------+-----------+-----------+

m1 | 292 0 |

m4 | 0 28 |

-----+-----------+-----------+

m1 | 291 22 | 313 0 |

m2 | 1 6 | 0 7 |

-----+-----------+-----------+-----------+

| m1 m4 | m1 m2 | m1 m2 | m1 m2 | m1 m2 | m1 m7 m23 | m1 m2 | m1 m2 | m1 m2 | m1 m2 |

| m1 m4 | m1 m2 | m1 m2 | m1 m2 | m1 m2 | m1 m7 m23 | m1 m2 | m1 m2 | m1 m2 | m1 m2 |

-----+-----------+-----------+-----------+-----------+-----------+----------------+-----------+-----------+-----------+-----------+

m1 | 288 25 | 307 6 | 313 0 |

m2 | 4 3 | 6 1 | 0 7 |

-----+-----------+-----------+-----------+-----------+

m1 | 289 14 | 297 6 | 297 6 | 303 0 |

m2 | 3 14 | 16 1 | 16 1 | 0 17 |

-----+-----------+-----------+-----------+-----------+-----------+

m1 | 288 24 | 306 6 | 307 5 | 296 16 | 312 0 |

m2 | 4 4 | 7 1 | 6 2 | 7 1 | 0 8 |

-----+-----------+-----------+-----------+-----------+-----------+----------------+

m1 | 234 15 | 244 5 | 246 3 | 238 11 | 245 4 | 249 0 0 |

m7 | 35 7 | 42 0 | 41 1 | 36 6 | 41 1 | 0 42 0 |

m23 | 23 6 | 27 2 | 26 3 | 29 0 | 26 3 | 0 0 29 |

-----+-----------+-----------+-----------+-----------+-----------+----------------+-----------+

m1 | 264 21 | 278 7 | 280 5 | 273 12 | 277 8 | 247 11 27 | 285 0 |

m2 | 28 7 | 35 0 | 33 2 | 30 5 | 35 0 | 2 31 2 | 0 35 |

-----+-----------+-----------+-----------+-----------+-----------+----------------+-----------+-----------+

m1 | 284 28 | 305 7 | 305 7 | 295 17 | 304 8 | 246 38 28 | 280 32 | 312 0 |

m2 | 8 0 | 8 0 | 8 0 | 8 0 | 8 0 | 3 4 1 | 5 3 | 0 8 |

-----+-----------+-----------+-----------+-----------+-----------+----------------+-----------+-----------+-----------+

m1 | 257 23 | 274 6 | 276 4 | 263 17 | 274 6 | 241 34 5 | 253 27 | 280 0 | 280 0 |

m2 | 35 5 | 39 1 | 37 3 | 40 0 | 38 2 | 8 8 24 | 32 8 | 32 8 | 0 40 |

-----+-----------+-----------+-----------+-----------+-----------+----------------+-----------+-----------+-----------+-----------+

m1 | 282 28 | 303 7 | 303 7 | 293 17 | 302 8 | 244 39 27 | 276 34 | 304 6 | 276 34 | 310 0 |

m2 | 10 0 | 10 0 | 10 0 | 10 0 | 10 0 | 5 3 2 | 9 1 | 8 2 | 4 6 | 0 10 |

-----+-----------+-----------+-----------+-----------+-----------+----------------+-----------+-----------+-----------+-----------+

| m1 m4 | m1 m2 | m1 m2 | m1 m2 | m1 m2 | m1 m7 m23 | m1 m2 | m1 m2 | m1 m2 | m1 m2 |
